# A Practical and Total Synthesis of Pasireotide: Synthesis of Cyclic Hexapeptide via a Three-Component Condensation

**DOI:** 10.3390/molecules24112185

**Published:** 2019-06-11

**Authors:** Chunying Ma, Miao Chen, Weiming Chu, Jiayi Tao, Delong Kong, Mengmeng Zhang, Wenhua Feng

**Affiliations:** Department of New Drug Research and Development, Institute of Materia Medical, Chinese Academy of Medical Sciences & Peking Union Medical College, Beijing 100050, China; machunying@imm.ac.cn (C.M.); chenmiao232@outlook.com (M.C.); chuweiming@imm.ac.cn (W.C.); taojiayi@imm.ac.cn (J.T.); kongdl@imm.ac.cn (D.K.); zhangmengmeng@imm.ac.cn (M.Z.)

**Keywords:** pasireotide, liquid-phase, synthesis, BSA/NHS, cyclic hexapeptide

## Abstract

Pasireotide is a multi-receptor ligand somatostatin analogue approved for medical treatment of Cushing’s disease and acromegaly. The liquid-phase total synthesis of pasireotide-a 18-membered cyclic hexapeptide-was achieved by the 3 + 2 + 1 strategy, and the Pro^1^-Phe^6^ peptide bond was selected as the final cyclization position. Two key fragments were simply synthesized using *N*,*O*-bis(trimethylsilyl)acetamide/*N*-hydroxysuccinimide ester (BSA/NHS) as coupling agents, and processes of the two key fragments were simple without any chromatographic purification. The current synthesis method is easily scalable and produces the target peptide with an overall yield of 15%.

## 1. Introduction

**Figure 1:**
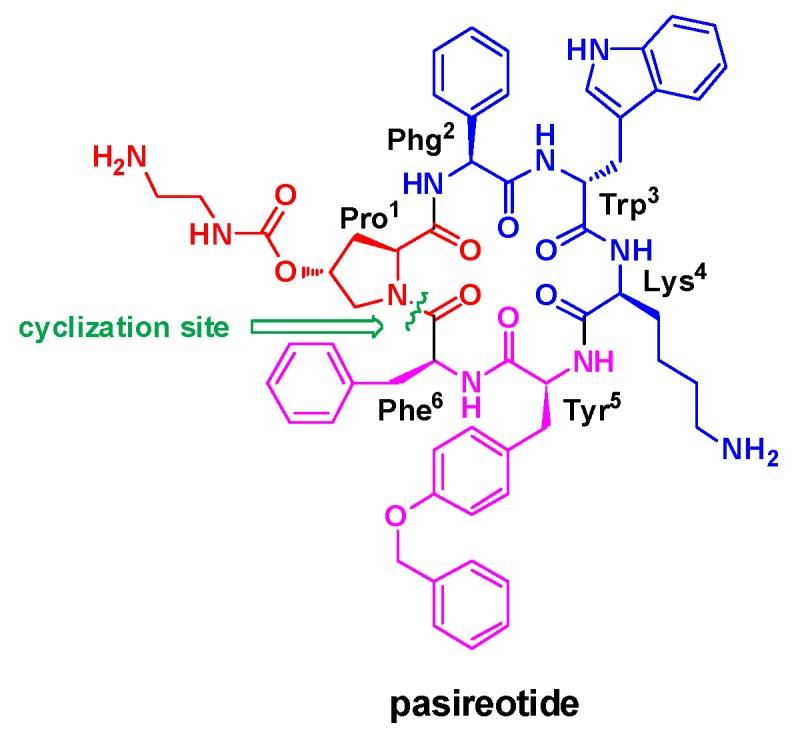
Structure of pasireotide.

Pasireotide (SOM230, Signifor) is a multi-receptor ligand somatostatin analogue (SSA) developed as the successor of the first-generation SSAs. Clinically, pasireotide is recommended for the treatment of patients with Cushing’s disease in whom surgery was unsuccessful, and patients with acromegaly who either remain uncontrolled after surgical therapy or in whom tumor resection is not possible [1,2,3,4]. Structurally, it belongs to cyclic hexapeptide featuring six amino acid moieties, including one proline (Pro), one phenylglycine (Phg), one tryptophane (Trp), one lysine (Lys), one tyrosine (Tyr), and one phenylalanine (Phe) (Figure 1). Due to its clinical attractiveness and structural unique, the synthesis of pasireotide has attracted increasing research interest of organic chemists from both academic and industrial laboratories. 

A variety of synthetic process for pasireotide has been developed in recent years [5,6,7,8,9,10,11,12]. In most of these reports, the synthesis routine was performed by solid-phase chemistry, using a Fmoc/tBu protecting scheme and resin as solid support. In solid-phase chemistry, significant shortcomings such as lack of scalability, inadequate in-process controls and low purity of the final products still hamper the development of scale-up synthesis [13]. Alternative approaches consisting of solution-phase reactions have also been developed to ensure well-established isolation, characterization, and purification protocols of the intermediate products [14]. In fact, most peptide-based pharmaceuticals are still synthesized using solution-phase chemistry, preferably employed for small- to medium-sized peptides [15], and pharmaceuticals containing as few as three amino acids (e.g., thyrotropin releasing hormone) and up to 32 residues (e.g., calcitonin) have been synthesized in solution-phase for clinical use [16]. 

**Scheme 1:**
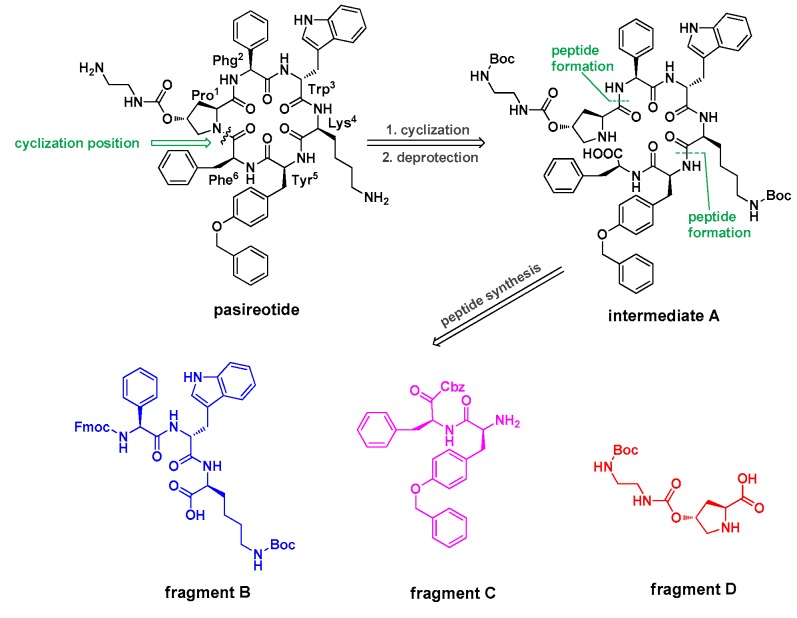
Structure of pasireotide A and our retrosynthetic analysis.

In liquid-phase method, the reported total synthesis of pasireotide was based on a linear strategy in which each amino acid was connected in turn [6]; however, the linear strategy increased the risk of low yield and time consumption. In this regard, the synthesis of pasireotide was conducted by a 3 + 2 + 1 condensation strategy in solution and was expected to increase the yield; and was easily handled in our current study. Based on the structure of pasireotide, the synthesis process comprises a cyclisation step of a corresponding linear peptide in protected form, and there are six cyclisation possibilities in it. According to our experience with peptide synthesis, the cyclisation between amino acids Pro^1^ and Phe^6^ is particularly preferred in view of the reduced steric hindrance of these two amino acid moieties relative to other residues in the molecule [17]. In this regard, the corresponding linear peptide was obtained by the 3 + 2 + 1 strategy and disconnected into three fragments, the tripeptide fragment B, dipeptide fragment C, and amino acid fragment D, based on the retrosynthetic analysis as outlined in Scheme 1. Herein, we reported detailed synthesis of pasireotide, the synthesis of building blocks, as well as the cleavage pattern of this product. Additionally, a simplified and easily-handled method using *N*,*O*-bis(trimethylsilyl)acetamide/*N*-hydroxysuccinimide ester (BSA/NHS) as coupling agents for peptide synthesis was carried out in the current study [18]. Additionally, the coupling reaction using BSA/NHS occurred without additional acid/base required, and all byproducts, as well as excessive reactants, were water soluble or hydrolysable and easily eliminated through water-washing at the purification stage.

## 2. Results and Discussion

As outlined in the synthetic strategy (Scheme 1), intermediate A could be transformed into pasireotide via cyclisation and deprotection of the amino groups. Additionally, protected key fragments B, C, and D could be prepared effectively from commercially-available protected amino acid moieties. The most important step was the construction of the peptide bond under mild conditions with BSA/NHS as the coupling reagent and no additional acid/base required.

**Scheme 2:**
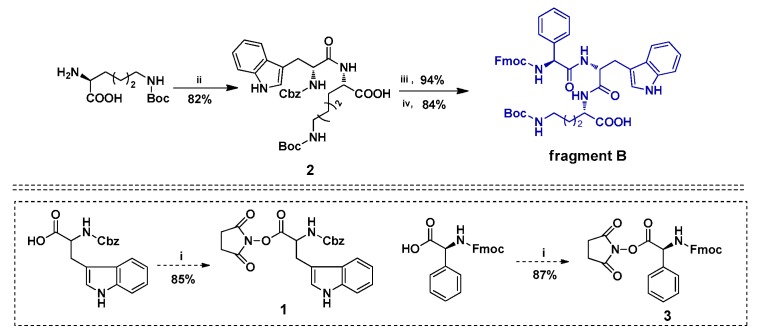
Synthesis of fragment B. Reaction condition and reagents: (i) *N*-hydroxysuccinimide(1.0 equiv.), *N*,*N′*-Dicyclohexylcarbodiimide (DCC)(1.1 equiv.), Tetrahydrofuran (THF); (ii) 1(0.83 equiv.), BSA(1.75 equiv.), Dichloromethane (CH_2_Cl_2_); (iii) Palladium over activated charcoal (Pd/C_10%_) (10%, *w*/*w*), H_2_, Methanol (MeOH); (iv) 3(0.83 equiv.), BSA(1.75 equiv.), THF.

**Figure 2:**
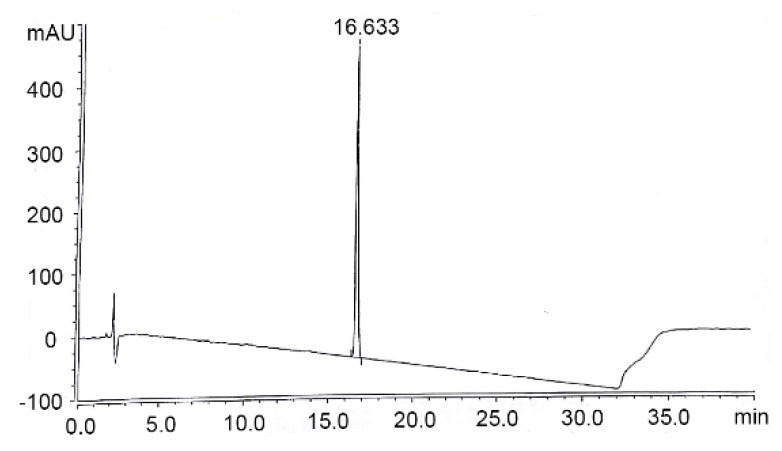
HPLC spectra of fragment B.

The fragment B was synthesized in five steps using intermediates 1 and 3 (Scheme 2). Firstly, the commercially-available *Ne*-Boc-l-lysine was coupled with *N*-Cbz-Trp NHS ester 1 in the presence of BSA to give compound **2** in 82% yield. Subsequently, compound **2** was treated with hydrogen (H_2_) in the presence of Pd/C, which was deprotected at the amino group to obtain amine intermediate in 94% yield. Then, the amine intermediate was treated with Fmoc-Phg NHS ester 3 in the presence of coupling reagent BSA to give fragment B in 84% yield. The high performance liquid chromatography (HPLC) analysis of fragment B is shown in Figure 2.

In the synthesis of fragment B, the use of BSA in combination with NHS ester was found to be effective for the coupling reaction (Scheme 2, steps i and iii), in which no additional acid/base was required, and all byproducts and excessive reactants were water soluble or hydrolysable and easily eliminated through water washing at the purification stage. Taking the synthesis of compound **2** for example, the coupling conditions and purification process were optimized, as shown in Table 1 (entries 1–4). When no BSA was added, the coupling product 2 was hardly detected (entry 1), and the coupling efficiency and yield were optimal when 1.2 equiv. of *Ne*-Boc-l-lysine reacted with 2.1 equiv. of BSA first in CH_2_Cl_2_ at room temperature, followed by the addition of 1.0 equiv. *N*-Cbz-Trp NHS ester 2 (entry 2). Significantly, the ratio of BSA was so important that either insufficient or excessive BSA would reduce the coupling yield (entries 3 and 4). Additionally, good yields of fragment B can be obtained in the ratio 2.1/1.2/1.0 of BSA/AA/NHS ester in THF or CH_2_Cl_2_ solvent (entries 5 and 8), respectively.

**Scheme 3:**
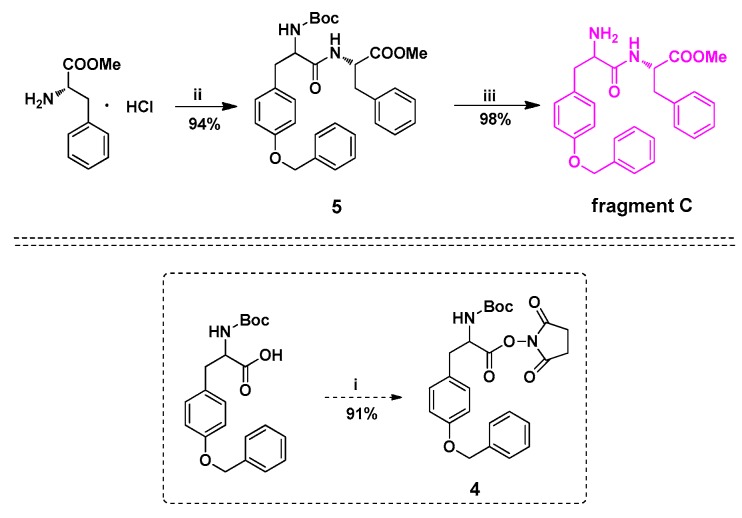
Synthesis of fragment C. Reaction condition and reagents: (i) *N*-hydroxysuccinimide(1.0 equiv.), DCC(1.1 equiv.), THF; (ii) 4(1.0 equiv.), TEA(1.0 equiv.), THF; (iii) TFA(10.0 equiv.), CH_2_Cl_2._

**Figure 3:**
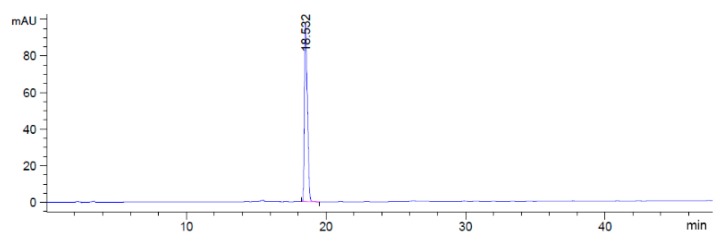
HPLC spectra of fragment C.

The fragment C was synthesized in three steps (Scheme 3). Firstly, *N*-Boc-Tyr NHS ester 4 was treated with commercially-available phenylalanine methyl ester in the presence of triethylamine (TEA) to obtain compound **5** in 94% yield. Then, compound **5** was treated with trifluoroacetic acid (TFA) to deprotect the amino group, giving fragment C in 98% yield. The HPLC analysis of fragment C is shown in Figure 3.

**Scheme 4:**
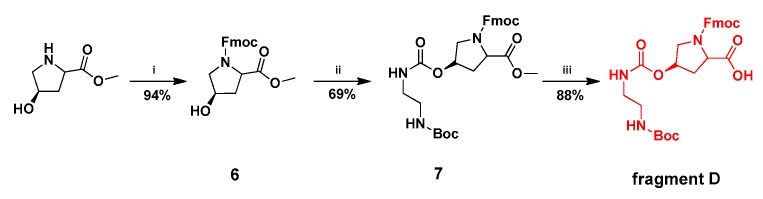
Synthesis of fragment D. Reaction condition and reagents: (i) Fmoc-OSu(1.0 equiv.), 1N Na_2_CO_3_ (1.0 equiv.), Dioxane/H_2_O; (ii) triphosgene(0.6 equiv.), *N*-boc-ethylenediamine(5.0 equiv.), THF; (iii) 1M NaOH(aq) (2.0 equiv.), Fmoc-OSu(1.0 equiv.), Dioxane.

**Figure 4:**
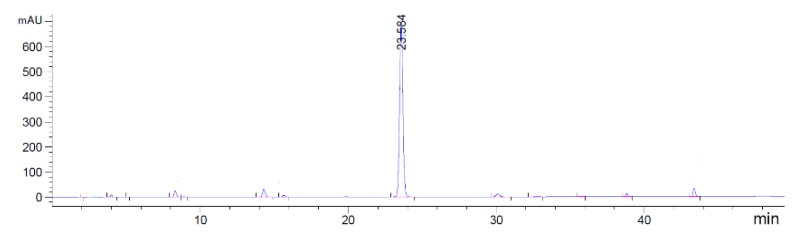
HPLC spectra of fragment D.

The synthesis of fragment D was accomplished in three steps as shown in Scheme 4. The commercially-available l-hydroxyproline methyl ester was protected by Fmoc *N*-hydroxysuccinimide ester (Fmoc-OSu) in the presence of Na_2_CO_3_, providing compound **6** in 94% yield. Condensing of 6 with triphosgene gas followed by *N*-boc-ethylenediamine procedure, produced compound **7** in 69% yield. Removal of methyl ester protecting group of 7 by treating with sodium hydroxide (NaOH) gave fragment D in 88% yield. The HPLC analysis of fragment D is shown in Figure 4.

**Scheme 5:**
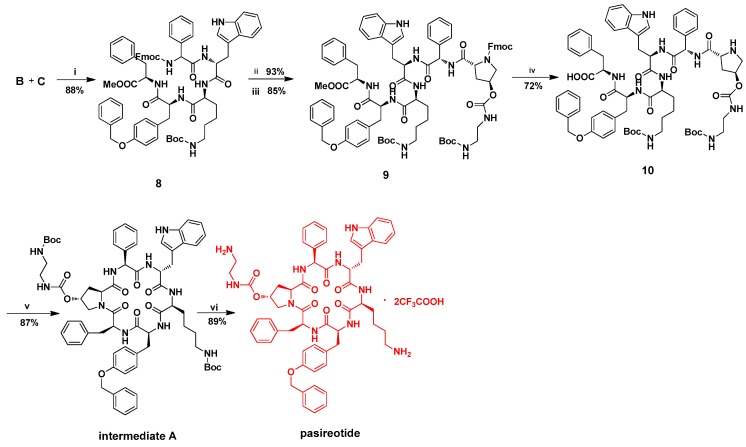
Synthesis of pasireotide. Reaction condition and reagents: (i) *O*-(1H-Benzo-1,2,3-Triazol-1-yl)-*N*,*N*,*N*′,*N*′-Tetramethyl-Uronium Tetrafluoroborate (TBTU) (1.5 equiv.), 4-methylmorpholine(2.0 equiv.), THF; (ii) piperidine(50.0 equiv.), THF; (iii) fragment D(1.0 equiv.), TBTU(1.5 equiv.), 4-methylmorpholine(2.0 equiv.), THF; (iv) piperidine(50.0 equiv.), THF; Lithium bromide (LiBr) (5.0 equiv.), NaOH(5.0 equiv.); (v) *O*-(7-Azabenzotriazol-1-yl)-*N*,*N*,*N*′,*N*′-Tetramethyluronium Hexafluorophosphate (HATU) (2.5 equiv.), 1-Hydroxybenzotriazole (HOBt) (2.5 equiv.) anhydrous, *N*,*N*-Dimethylformamide (DMF); (vi) TFA(200 equiv.).

With the two key fragments B and C in hand, firstly we were ready to assemble intermediate **8** by choosing the formation of the Lys^4^-Tyr^5^ amide bond in the presence of TBTU/4-methylmorpholine (Scheme 5, step i). Subsequently, removal of the Fmoc protecting group in compound **8**, by treating with piperidine, was followed by condensing with fragment D in the presence of TBTU/4-methylmorpholine, yielding compound **9** in 85% yield (Scheme 5, steps ii and iii).

Then, hydrolysis of the methyl ester protecting group of compound **9** afforded the carboxylic acid of compound **10** (Scheme 5, step iv). It is well-known that hydrolysis of ester is under alkaline conditions which has a significant impact on the conformation of peptide [19,20,21,22,23,24]. However, the macrocyclization step is critical in the synthesis of cyclic peptide, and may depend on the peptide sequence, structural constraints, and the resulting ring [25,26,27]. In the synthesis of intermediate A, a series of optimization experiments were carried out to achieve an acceptable yield (Table S1, Supporting Information).

After attempting various alkaline conditions, we found that the best result could be achieved by using LiBr/NaOH conditions (Table 2, entry 3), leading to compound **10** in 72% yield with no epimerization product according to the HPLC analysis (Figure S31, Supporting Information). In contrast, the hydrolysis of compound **9** under NaOH solutions (Table 2, entry 1) resulted in epimerization of compound **10** according to the HPLC analysis (Figure S32, Supporting Information).

After the liner pentapeptide 10 was obtained, the macrocyclization was accomplished by using HATU/ anhydrous HOBt in DMF to generate the key intermediate A in 87% yield (Scheme 5, step v). Finally, treatment of precursor A with TFA furnished the expected target pasireotide A in 89% yield (Scheme 5, step vi). Finally, the purification was carried out on recycling preparative reversed-phase HPLC (RP-HPLC) using a Jaigel Polyamine column and 1:1 ratio of water:acetonitrile as the solvent system (Figure S33, Supporting Information). The MS spectrum of the major peak in HPLC showed the molecular ion peak and doubly protonated peak of pasireotide A at *m*/*z* 1047.50 and 524.25, respectively.

**Figure 5:**
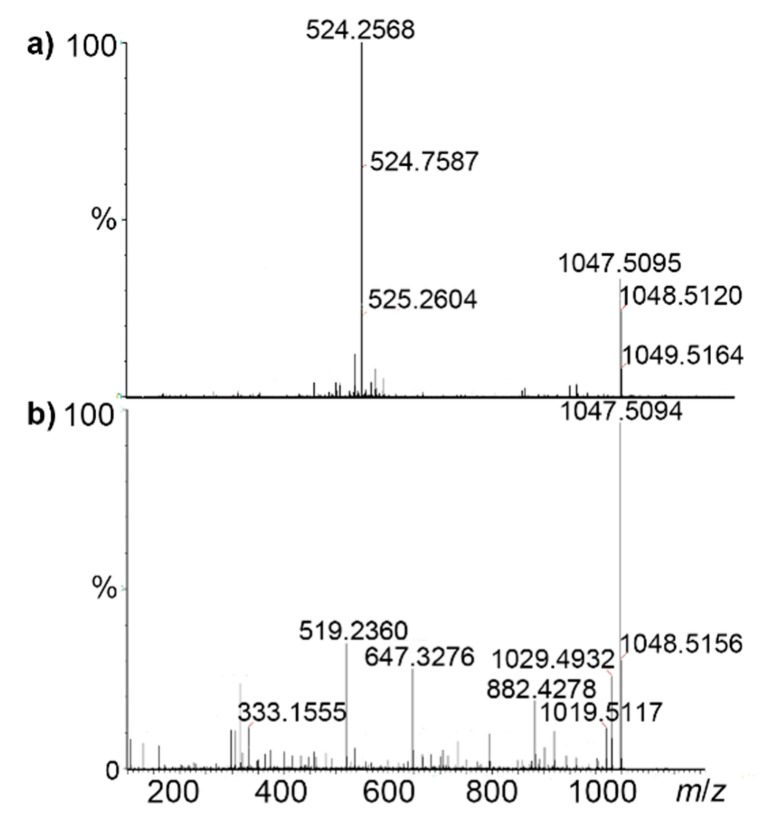
(**a**) Mass Spectrometry (MS) and (**b**) MS/MS spectra of protonated pasireotide.

**Scheme 6:**
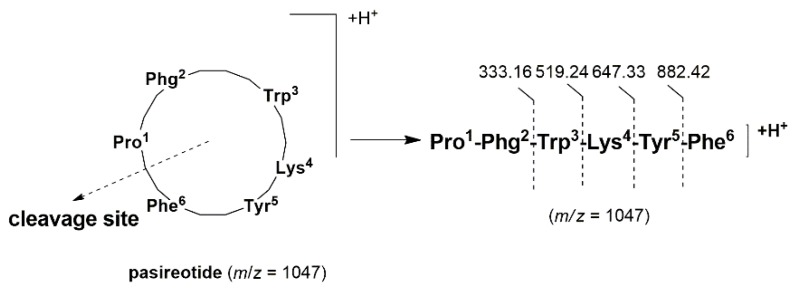
Cleavage site of protonated pasireotide by MS/MS spectrum.

The high resolution mass spectrometry (HRMS) of pasireotide displayed a protonated molecular ion peak [M + H]^+^ at *m*/*z* 1047.5094, corresponding to the molecular formula C_58_H_67_N_10_O_9_ (calculated 1047.5092), as well as doubly protonated peak [M + 2H]^+^ at *m*/*z* 524.2568 (calculated 524.2585) (Figure 5a). Under collision-induced decomposition (CID) experiment [28] (MS/MS), the protonated molecular ion peak at *m*/*z* 1047.5094 produced ions at *m*/*z* 1029.4932[M + H − H_2_O]^+^ and at *m*/*z* 1019.5117 [M + H − CO]^+^ (Figure 5b). It is reported that an amide bond with a higher proton affinity may be more readily opened due to the basicities of the amide nitrogen [29]. What is more, this is the case with proline-containing cyclic peptides when collision-activated under energy conditions; they afforded selectivity by undergoing selective ring cleavage at the proline residue [30]. Hence, the linear peptide [Pro^1^-Phg^2^-Trp^3^-Lys^4^-Tyr^5^-Phe^6^]H^+^ was produced by the cleavage of Pro^1^-Phe^6^ amide bond (Scheme 6) and subsequently produced a main series of adjacent peaks at *m*/*z* 882.4278, 647.3276, 519.2360, and 333.1555 (Figure 2b) corresponding to the successive loss of Phe^6^, Tyr^5^, Lys^4^, and Trp^3^, respectively (Scheme 6).

The structure of pasireotide was further confirmed through nuclear magnetic resonance spectroscopy (NMR) (see Experimental Section), and it was found to be spectroscopically identical with that of the standard product.

## 3. Conclusion

In summary, we successfully applied a liquid-phase synthetic route to access pasireotide, a cyclic hexapeptide used to treat Cushing’s disease, through decentralized 3 + 2 + 1 strategy, and the overall yield of pasireotide was about 15% in our study, which is comparable to that reported 20% in solid-phase chemistry [5]. Our synthesis formed the Pro^1^-Phe^6^ peptide bond as the final macrocyclization site, and three key fragments can be easily obtained without any chromatographic purification. The spectroscopic data of all of the synthetic products were in good agreement with those reported for the product.

## 4. Experimental Section

**General Experimental Procedures.** All reactions were performed under a nitrogen atmosphere using anhydrous techniques unless otherwise noted. ^1^H and ^13^C-NMR on a Varian Mercury 500 spectrometer were recorded in DMSO-d_6_ or CDCl_3_. Chemical shifts were reported in δ(ppm) units relative to the internal standard tetramethylsilane (TMS). All the reactions were monitored by thin layer chromatography (TLC) analysis on pre-coated silica gel G plates at 254 nm under UV lamp or HPLC analysis.

**Chromatographic Methods.** Analysis for fragments B, C, and D was achieved on the Agilent model 1260-DAD high-performance liquid chromatographsystem along with the detective wavelength of 220, 225, and 280 nm. The mobile phase consisted of acetonitrile (A) and deionized water (B), both with 0.1% TFA (trifluoroacetic acid) (vol./vol.), using gradient elution. The flow rate was 1.0 mL·min^−1^, and the column temperature was maintained at 25 °C. The concrete gradient elution conditions are displayed in Table S2. Analysis for compound **10** was achieved on the Waters UPLC, using gradient elution. The preparative column chromatography for crude pasireotide was performed on SHIMADZU Shim-pack PRC-ODS (H) (20 × 250 mm), using gradient elution.

**Materials.** Protected amino acids, PyBop, and anhydrous HOBt were purchased from Aldrich. All other reagents and chemicals were purchased from Beijing Chemistry Works (Beijing, Chnia), and were used without further purification. Anhydrous organic solvents were obtained either from Aldrich or distilled from the drying agents: Na or CaH_2_. All reactions were carried out under a N_2_ atmosphere employing oven- or flame-dried glassware. All solvents were either distilled or obtained from passing through activated alumina. Acetonitrile and trifluoroacetic acid (HPLC grade) were purchased from Sigma-Aldrich (Milwaukee, Germany). Ultrapure water was purchased from Hangzhou Wahaha Co., Ltd. (Hangzhou, China)

**Synthesis of Compound 1.** To a stirred solution of *N*-Cbz-d-Trp (10.0 g, 29.6 mmol) in THF (100 mL) was added *N*-hydroxysuccinimide (3.4 g, 29.6 mmol) at 0 °C. The resulting solution was then added dropwise to a solution of *N*,*N*’-dicyclohexylcarbodiimide (DCC) (6.7 g, 32.6 mmol) in THF (20 mL). The mixture was stirred at room temperature for 10 h. After the reaction was completed, DCU was removed by filtration through celite and the crude product was obtained by removing the solvent under vacuum. Pure compound **1**, as a slight yellow solid, was obtained after recrystallization in isopropyl alcohol (80% yield). HRMS: *m*/*z* Calcd. 436.1509 For C_23_H_22_N_3_O_6_, Found 436.1504; ^1^H-NMR(500 MHz, DMSO-d_6_) δ/ppm *m*/*z* 10.94(s, 1H), 8.14(d, *J* = 8.0 Hz, 1H), 7.56(d, *J* = 8.0 Hz, 1H), 7.38–7.26(m, 6H), 7.09(t, *J* = 16.0 Hz, 2H), 7.04(t, *J* = 12.0 Hz, 1H), 4.99(q, *J* = 12.6 Hz, 2H), 4.69–4.63(m, 1H), 3.35(dd, *J* = 4.0, 16.0 Hz, 1H), 3.17(dd, *J* = 12.0, 8.0 Hz, 1H), 2.84(s, 4H); ^13^C-NMR(125 MHz, DMSO-d_6_) δ/ppm = 170.74, 169.08,156.55,137.28, 136.80, 129.02, 128.50, 128.27, 127.45, 124.97, 121.78, 119.31, 118.51, 112.25, 109.38, 60.44, 66.36, 27.71, 26.18.

**Synthesis of Compound 2.** To a solution of *Ne*-Boc-l-lysine (5.0 g, 20.3 mmol) in CH_2_Cl_2_ (50 mL) was added, dropwise, BSA (7.2 g, 35.5 mmol) at 0 °C followed by compound **1** (7.3 g, 16.8 mmol) in CH_2_Cl_2_ (50 mL). The mixture was stirred at room temperature for 12 h and then concentrated in vacuo. The residue was dissolved in ether (150 mL) and extracted with 5% sodium hydrocarbonate (NaHCO_3_) solution (50 mL × 3). The combined water layer was acidified with citric acid to pH 6–7 and extracted with EtOAc (100 mL × 3). The combined organic layers were washed with brine, dried over Na_2_SO_4_, and the solvent was removed by a rotary evaporator, under reduced pressure, to yield 2 as a yellow power in 82% yield. HRMS: *m*/*z* Calcd. 567.2918 For C_30_H_39_N_4_O_7_, Found 567.2915; ^1^H-NMR(500 MHz, DMSO-d_6_) δ/ppm = 12.58(s, 1H), 10.79(s, 1H), 8.24(d, *J* = 8.0 Hz, 1H), 7.66(d, *J* = 8.0 Hz, 1H), 7.35–7.28(m, 4H), 7.25–7.24(d, *J* = 4.0 Hz, 1H), 7.15(s, 1H), 7.05(t, *J* = 4.0 Hz, 1H), 6.96(t, *J* = 7.5 Hz, 1H), 6.74(s, 1H), 4.94(s, 2H), 4.38(td, *J* = 8.0, 12.0 Hz, 1H), 4.15(q, *J* = 7.6 Hz, 1H), 3.07(dd, *J* = 8.0, 16.0 Hz, 1H), 2.94–2.88(m, 1H), 2.85(d, *J* = 4.0 Hz, 2H), 1.65–1.63(m, 1H), 1.55–1.51(m, 1H), 1.35–1.30(m, 11H), 1.17(t, *J* = 8.0 Hz, 2H); ^13^C-NMR(125 MHz, DMSO-d_6_) δ/ppm = 174.31, 172.36, 156.21, 137.68, 136.68, 128.95, 128.30, 128.08, 127.92, 124.55, 121.44, 119.27, 118.79, 111.89, 110.74, 77.99, 65.83, 60.43, 56.06, 31.68, 29.77, 28.91, 23.19, 21.43, 14.74.

**Synthesis of Compound 3.** To a stirred solution of Fmoc-l-phenylglycine (15.0 g, 40.0 mmol) in THF (150 mL) was added *N*-hydroxysuccinimide (4.6 g, 40.0 mmol), dropwise, at 0 °C followed by DCC (9.1 g, 44.0 mmol) in THF dropwise at 5 °C. The mixture was stirred at room temperature for 6 h. After the reaction was completed, DCU was removed by filtration through celite and the crude product was obtained by removing the solvent under vacuum. Pure compound **3**, as a white solid, was obtained after recrystallization in EtOAc (87% yield). HRMS: *m*/*z* Calcd. 470.1478 For C_27_H_23_N_2_O_6_, Found 470.1474; ^1^H-NMR(500 MHz, CDCl_3_) δ/ppm = 7.74–7.75(d, 2H, *J* = 5.0 Hz), 7.56(s, 2H), 7.37–7.46(m, 7H), 7.28(s, 2H), 5.79–5.80(d, 1H, *J* = 5.0 Hz), 5.68(s, 1H), 4.42–4.45(t, 2H, *J* = 7.5 Hz), 4.21(s, 1H), 2.78(s, 4H); ^13^C-NMR(125 MHz, CDCl_3_) δ/ppm = 168.33, 166.91, 155.22, 143.76, 143.57, 141.30, 134.41, 129.40, 129.31, 127.75, 127.62, 127.12, 125.07, 120.00, 67.45, 56.44, 47.07, 25.55.

**Synthesis of fragment B.** To a stirred solution of 2 (5.0 g, 8.8 mmol) in MeOH (50 mL) was added Pd/C_10%_ (0.5 g). A hydrogen atmosphere (1 atm) was applied and the mixture was stirred at room temperature for 5 h. The mixture was filtered through celite and the filtrate was concentrated in vacuo. Next, 5% NaHCO_3_ solution (30 mL) was added to dissolve the concentrated filtrate and then citric acid was added to adjust the pH to ≈ 6. After that, a white solid precipitated out and was collected by filtration to afford amine intermediate in 94%. HRMS: *m*/*z* Calcd. 433.2451 For C_25_H_33_N_4_O_5_, Found 433.2453. To a solution of the above amine (3.6 g, 8.3 mmol) in dry THF (30 mL) at 0 °C was added BSA (2.9 g, 14.5 mmol), dropwise, and the mixture was stirred for 2 h at room temperature, after which 3 (3.0 g, 6.9 mmol) in THF (20 mL) was added, dropwise, and the resulting mixture was stirred for 10 h at room temperature. After the reaction was completed, the solvents were evaporated. The residue was then dissolved in ether (50 mL) and extracted with 5% NaHCO_3_ solution (50 mL × 3). The combined water layer was acidified with citric acid to pH 6–7 and extracted with EtOAc (100 mL × 3). The combined organic layers were washed with brine, dried over Na_2_SO_4_, and filtered through celite. The organic solution was evaporated in vacuo and 4.57 g of fragment B was obtained after recrystallization in MeOH (84% yield). HRMS: *m*/*z* Calcd. 788.3659 For C_45_H_50_N_5_O_8_, Found 788.3650; ^1^H-NMR(500 MHz, DMSO-d_6_) δ/ppm = 12.53(s, 1H), 10.68(d, *J* = 2.4 Hz, 1H), 8.50(d, *J* = 8.1 Hz, 1H), 8.25(d, *J* = 7.8 Hz, 1H), 8.02(d, *J* = 8.5 Hz, 1H), 7.86(d, *J* = 7.5 Hz, 2H), 7.75(dd, *J* = 11.3, 7.6 Hz, 2H), 7.55(d, *J* = 7.9 Hz, 1H), 7.39(td, *J* = 7.4, 3.0 Hz, 2H), 7.31–7.17(m, 7H), 7.01(t, *J* = 7.4 Hz, 1H), 6.91(s, 1H), 6.71(t, *J* = 5.8 Hz, 1H), 5.36(d, *J* = 8.4 Hz, 1H), 4.57(q, *J* = 8.1 Hz, 1H), 4.22(dd, *J* = 13.5, 7.1 Hz, 2H), 3.04(dd, *J* = 14.5, 5.2 Hz, 1H), 2.88(d, *J* = 9.8 Hz, 1H), 2.82(q, *J* = 6.7, 6.1 Hz, 2H), 1.58(s, 1H), 1.49(t, *J* = 7.5 Hz, 1H), 1.33(s, 9H), 1.26(q, *J* = 7.2 Hz, 2H), 1.09(dt, *J* = 13.9, 7.6 Hz, 2H); ^13^C-NMR(125 MHz, CDCl_3_) δ/ppm = 174.25, 171.87, 170.20, 156.42, 156.21, 144.60, 144.32, 141.32, 138.99, 136.62, 128.70, 128.29, 127.94, 127.72, 126.25, 126.12, 124.52, 121.36, 120.73, 119.08, 118.74, 111.81, 110.15, 77.98, 66.67, 58.49, 54.19, 52.61, 47.23, 31.59, 30.58, 29.74, 28.91, 23.19.

**Synthesis of Compound 4.** To a stirred solution of *N*-Boc-*O*-benzyl-d-tyrosine (10.0 g, 27.0 mmol) in dry THF (100 mL) was added *N*-hydroxysuccinimide (3.1 g, 27.0 mmol) at 0 °C. The resulting solution was then added a solution of DCC (6.1 g, 29.7 mmol) in THF, dropwise, at 5 °C. The mixture was stirred at room temperature for 4 h and monitored by TLC. After the reaction was completed, DCU was removed by filtration through celite. The solvent was evaporated off, after which 11.5 g of 4 was obtained after recrystallization in EtOAc in 91% yield. HRMS: *m*/*z* Calcd. 469.1975 For C_25_H_29_N_2_O_7_, Found 469.1971; ^1^H-NMR(500 MHz, CDCl_3_) δ/ppm = 7.41–7.42(d, 2H, *J* = 5.0 Hz), 7.37–7.39(t, 2H, *J* = 5.0 Hz), 7.20–7.21(d, 2H, *J* = 5.0 Hz), 6.92–6.94(d, 2H, *J* = 10.0 Hz), 5.04(s, 2H), 4.89(s, 1H), 3.13–3.25(m, 2H), 2.84(s, 4H), 1.42(s, 9H); ^13^C-NMR(125 MHz, CDCl_3_) δ/ppm = 168.65, 167.77, 158.18, 154.62, 136.97, 130.82, 128.58, 127.96, 127.47, 126.86, 80.52, 70.00, 52.71, 37.28, 28.24, 25.59.

**Synthesis of Compound 5.** To a stirred solution of phenylalanine methyl ester hydrochloride (5 g, 23.3 mmol) in dry THF (50 mL) was added trimethylamine (2.4 g, 23.3 mmol), dropwise, followed by addition of 4 (10.9 g, 23.3 mmol) in THF (20 mL). The mixture was stirred at room temperature for 5 h and then concentrated in vacuo. The residue was dissolved in EtOAc (100 mL) and washed with 5% NaHCO_3_ solution (100 mL × 3), 5% citric acid solution (100 mL × 3), brine, and dried over Na_2_SO_4_. Filtration followed by concentration in vacuo afforded 11.6 g of 5, as a white solid, in 94% yield. HRMS: *m*/*z* Calcd. 533.2652 For C_31_H_37_N_2_O_6_, Found 533.2647; ^1^H-NMR(500 MHz, CDCl_3_) δ/ppm = 7.40–7.42(d, 2H, *J* = 10.0 Hz), 7.35–7.38(t, 2H, *J* = 7.50 Hz), 7.31–7.32(d, 1H, *J* = 5.0 Hz), 7.22–7.25(t, 3H, *J* = 7.50 Hz), 7.09–7.10(d, 2H, *J* = 5.0 Hz), 6.97–6.99(d, 2H, *J* = 10.0 Hz), 6.88–6.89(d, 2H, *J* = 5.0 Hz), 6.27(s, 1H), 5.02(s, 2H), 4.95(s, 1H), 4.77(s, 1H), 4.82(s, 1H), 3.66(s, 3H), 2.96–3.05(m, 4H), 1.40(s, 9H); ^13^C-NMR(125 MHz, CDCl_3_) δ/ppm = 171.36, 170.81, 157.89, 136.98, 135.65, 130.42, 129.22, 128.58, 128.54, 127.96, 127.42, 127.11, 115.03, 80.16, 70.01, 55.80, 53.26, 52.25, 37.99, 37.43, 28.26.

**Synthesis of fragment C.** To a stirred solution of 5 (6.0 g, 11.6 mmol) in CH_2_Cl_2_ (25 mL) was added trifluoroacetic acid (TFA) (8.6 mL, 116 mmol), dropwise, at 5 °C. The mixture was stirred at room temperature for 4 h and monitored by TLC. The solvent was evaporated off, after which 5.9 g of fragment C was obtained after recrystallization in ether (98% yield). HRMS: *m*/*z* Calcd. 433.2127 For C_26_H_29_N_2_O_4_, Found 433.2124; ^1^H-NMR(500 MHz, CDCl_3_) δ/ppm = 8.97–8.99(d, 1H, *J* = 10.0 Hz), 8.10(s, 3H), 7.44–7.45(d, 2H, *J* = 5.0 Hz), 7.38–7.41(t, 2H, *J* = 7.5 Hz), 7.29–7.34(m, 3H), 7.22–7.25(t, 3H, *J* = 7.5 Hz), 7.17–7.18(d, 2H, *J* = 5.0 Hz), 6.96–6.98(d, 2H, *J* = 10.0 Hz), 5.08(s, 2H), 4.55–4.60(q, 1H, *J* = 8.3 Hz), 3.98(s, 1H), 3.61(s, 3H), 3.02–3.09(m, 2H), 2.55–3.00(dd, 1H, *J_1_* = 10.0, *J_2_* = 15.0Hz), 2.85–2.89(dd, 1H, *J_1_* = 5.0, *J_2_* = 15Hz); ^13^C-NMR(125 MHz, CDCl_3_) δ/ppm = 171.64, 168.76, 158.08, 137.58, 137.14, 131.12, 129.53, 128.89, 128.86, 128.09, 127.21, 127.15, 115.27, 69.63, 54.27, 53.76, 52.50, 37.10, 36.51.

**Synthesis of Compound 6.** To a stirred solution of l-hydroxyproline methyl ester (10.0 g, 69.9 mmol) in 1,4-dioxane/H_2_O (*v*:*v* = 2:1) was added Na_2_CO_3_ (7.3 g, 69.9 mmol), slowly, followed by Fmoc N-hydroxysuccinimide ester (Fmoc-OSu) (23.6 g, 69.9 mmol). The resulting solution was stirred at room temperature for 10 h and then concentrated in vacuo. The residue was dissolved in EtOAc (100 mL), washed with brine, dried over Na_2_SO_4_, and concentrated to yield 6, as a colorless oil, in 94% yield. HRMS: *m*/*z* Calcd. 368.1498 For C_21_H_22_NO_5_, Found 368.1494; ^1^H-NMR(500 MHz, CDCl_3_) δ/ppm = 7.80–7.79(d, *J* = 4 Hz, 2H), 7.66–7.58(m, 2H), 7.45–7.42(t, *J* = 6.0 Hz, 2H), 7.36–7.34(t, *J* = 4.0 Hz, 2H), 4.59–4.44(m, 3H), 4.41–4.35(m, 1H), 4.31–4.15(m, 1H), 3.79(s, 2H), 3.70–3.60(m, 3H), 2.74(s, 1H), 2.41–2.32(m, 1H), 2.15–2.13(m, 1H); ^13^C-NMR(125 MHz, CDCl_3_) δ/ppm = 173.16, 155.10, 144.09, 141.33, 127.74, 127.12, 125.15, 119.99, 70.08, 69.22, 67.64, 60.51, 57.98, 57.62, 55.31, 54.68, 52.38, 47.27, 39.33, 38.42.

**Synthesis of Compound 7.** To a stirred solution of triphosgene (3.2 g, 10.6 mmol) in dry THF (40 mL) was added 6 (6.5 g, 17.7 mmol) in THF (10 mL), slowly, and the mixture was stirred at room temperature for 2 h. Next, *N*-boc-ethylenediamine (14.2 g, 88.5 mmol) in THF (20 mL) and 4-dimethylaminopyridine (2.2 g, 17.7 mmol) was added. The resulting mixture was stirred at room temperature for 6 h. After the reaction was completed, the solvents were evaporated and then the residue was dissolved in EtOAc (100 mL), washed with 0.1 M HCl solution (100 mL × 3), brine (100 mL × 3), and dried over Na_2_SO_4_. The solvent was evaporated off, after which 6.7 g of **7** was obtained after recrystallization in EtOAc in 69% yield. HRMS: *m*/*z* Calcd. 576.2322 For C_29_H_35_N_3_O_8_Na, Found 576.2313; ^1^H-NMR(500 MHz, CDCl_3_) δ/ppm = 7.77(d, *J* = 8.0 Hz, 2H), 7.57(dd, *J* = 12.0, 4.0 Hz, 2H), 7.40(t, *J* = 8.0 Hz, 2H), 7.31(t, *J* = 6.0 Hz, 2H), 5.26(d, *J* = 20.0 Hz, 1H), 5.14(d, *J* = 20.0 Hz, 1H), 4.79(s, 1H), 4.51–4.47(m, 1H), 4.43–4.39(m, 1H), 4.34(t, *J* = 8.0 Hz, 1H), 4.29–4.15(m, 1H), 3.85–3.72(m, 4H), 3.66(s, 1H), 3.27(s, 4H), 2.46(dd, *J* = 12.0, 20.0 Hz, 2H), 2.22(s, 1H), 1.44(s, 9H); ^13^C-NMR(125 MHz, CDCl_3_) δ/ppm = 172.53, 155.78, 154.75, 143.91, 143.59, 141.29, 127.73, 127.08, 125.12, 120.00, 79.75, 73.13, 72.23, 67.73, 60.40, 52.43, 47.27, 28.35, 21.04, 14.20.

**Synthesis of fragment D.** To a stirred solution of 7 (5.0 g, 9.0 mmol) in dry MeOH (30 mL) was added NaOH solution (18.0 mmol, 10 mL), dropwise, at 0 °C and the mixture was stirred at room temperature for 2 h. Solvents were removed by vacuo and the residue was resolved in 1, 4-dioxane followed by addition of Fmoc-OSu (3.0 g, 9.0 mmol). The resulting mixture was stirred at room temperature for 6 h and solvents were evaporated, after which 4.2 g of fragment D was obtained in 88% yield. HRMS: *m*/*z* Calcd. 562.5667 For C_28_H_33_N_3_O_8_Na, Found 562.2167. ^1^H-NMR(500 MHz, DMSO-d_6_) δ/ppm = 7.93–7.89(t, *J* = 10.0 Hz, 1H)7.77–7.74(dd, *J_1_* = 10.0, *J_2_* = 45 Hz, 1H), 7.66(t, *J* = 7.5 Hz, 1H), 7.44(q, *J* = 6.6 Hz, 2H), 7.36(t, *J* = 5.0 Hz, 2H), 7.27(s, 1H), 6.85(s, 1H), 5.11(s, 1H), 4.28–4.19(m, 3H), 4.05(q, *J* = 10.0 Hz, 1H), 3.65–3.73(ddd, *J* = 27.8, 11.9, 5.3 Hz, 1H), 3.46–3.38(m, 1H), 3.43(s, 1H), 3.01(s, 4H), 2.25(s, 1H), 2.15(s, 1H), 1.39(s, 9H). ^13^C-NMR (125 MHz, DMSO-d_6_) δ/ppm = 171.00, 156.22, 155.12, 141.34, 140.04, 138.05, 129.57, 127.94, 122.05, 120.69, 78.25, 72.56, 67.37, 60.42, 52.99, 47.22, 28.87, 21.43, 14.74.

**Synthesis of Compound 8.** To a stirred solution of fragments B (3.5 g, 4.4 mmol) and C (1.4 g, 4.4 mmol) in dry THF (50 mL) was added TBTU (2.1 g, 6.6 mmol) and 4-methylmorpholine (0.9 g, 8.8 mmol), slowly. The mixture was stirred at room temperature for 6 h and solvents were removed by vacuo. The residue was dissolved in EtOAc (100 mL) and washed with 5% NaHCO_3_ solution (100 mL × 3), 5% citric acid solution (100 mL × 3), brine, and dried over Na_2_SO_4_. Solvents were evaporated off, after which 4.7 g of **8** was obtained after recrystallization in MeOH in 88% yield. HRMS: *m*/*z* Calcd. 1202.5603 For C_71_H_76_N_7_O_11_, Found 1202.5590; ^1^H-NMR(500 MHz, DMSO-d_6_) δ/ppm = 10.72(s, 1H), 8.64(d, *J* = 8.0 Hz, 1H), 8.27(d, *J* = 4.0 Hz, 1H), 8.17(d, *J* = 8.0 Hz, 1H), 7.97–7.91(dd, *J* = 16.0, 4.0 Hz, 1H), 7.85(d, *J* = 8.0 Hz, 2H), 7.74–7.68(dd, *J* = 16.0, 8.0 Hz, 2H), 7.53(d, *J* = 4.0 Hz, 1H), 7.37–7.33(m, 5H), 7.32–7.25(m, 7H), 7.23–7.17(m, 8H), 7.13(d, *J* = 8.0 Hz, 2H), 7.00(t, *J* = 6.0Hz, 1H), 6.93–6.89(m, 2H), 6.84(d, *J* = 8.0 Hz, 2H), 6.67(t, *J* = 6.0 Hz, 1H), 5.38(d, *J* = 8.0 Hz, 1H), 4.97(s, 2H), 4.48–4.45(m, 3H), 4.22–4.16(m, 3H), 4.09(s, 1H), 3.58–3.51(m, 1H), 3.47(s, 2H), 3.07–2.98(m, 3H), 2.94–2.86(m, 2H), 2.79–2.74(m, 3H), 1.48–1.43(m, 2H), 1.33(s, 9H), 1.20(s, 2H), 0.93(d, *J* = 8.0 Hz, 2H); ^13^C-NMR(125 MHz, DMSO-d_6_) δ/ppm = 171.88, 170.73, 157.52, 156.22, 144.66, 144.22, 141.30, 140.05, 138.97, 138.05, 137.77, 136.62, 130.88, 129.72, 129.61, 129.02, 128.90, 128.30, 127.98, 127.73, 127.62, 127.23, 126.20, 122.05, 120.70, 119.10, 118.83, 114.92, 111.83, 110.48, 110.00, 78.05, 69.71, 66.72, 58.40, 54.52, 53.49, 52.30, 47.17, 37.29, 35.75, 31.93, 29.87, 28.89, 27.19, 22.93.

**Synthesis of Compound 9.** To a stirred solution of 8 (4.0 g, 3.3 mmol) in dry THF (40 mL) was added piperidine (16.5 mL, 165 mmol), dropwise, at 0 °C and the mixture was stirred at room temperature for 40 min. Solvents were evaporated off, after which 3.0 g of amine intermediate was obtained after recrystallization in MeOH in 93% yield. HRMS: *m*/*z* Calcd. 980.4922 For C_56_H_66_N_7_O_9_, Found 980.4932. To a stirred solution of the above amine (1.7 g, 3.1 mmol) and fragment D (3.0 g, 3.1 mmol) in dry THF (50 mL) was added TBTU (1.5 g, 4.7 mmol) and 4-methylmorpholine (0.9 g, 9.3 mmol), slowly. The mixture was stirred at room temperature for 6 h and solvents were removed by vacuo. The residue was dissolved in EtOAc (100 mL) and washed with 5% NaHCO_3_ solution (100 mL × 3), 5% citric acid solution (100 mL × 3), brine, and dried over Na_2_SO_4_. Solvents were evaporated off, after which 3.96 g of **9** was obtained after recrystallization in MeOH in 85% yield. HRMS: *m*/*z* Calcd. 1501.7084 For C_84_H_97_N_10_O_16_, Found 1501.7087; ^1^H-NMR(500 MHz, DMSO-d_6_) δ/ppm = 10.69(dd, *J* = 27.1, 6.2 Hz, 1H), 8.54(dd, *J* = 42.4, 12.0 Hz, 2H), 8.36(dd, *J* = 21.2, 8.6 Hz, 1H), 8.08(dd, *J* = 19.8, 9.3 Hz, 1H), 7.96–7.90(m, 1H), 7.87(d, *J* = 5.0 Hz, 2H), 7.59(d, *J* = 7.8 Hz, 2H), 7.56–7.51(m, 1H), 7.42–7.11(m, 26H), 6.88(m, 2H), 5.58(dd, *J* = 39.8, 8.0 Hz, 1H), 4.96(m, 2H), 4.89–4.70(m, 1H), 4.49(s, 2H), 4.23(d, *J* = 9.8 Hz, 2H), 4.15(s, 1H), 4.08–4.00(m, 1H), 3.90(s, 1H), 3.80(t, *J* = 14.3 Hz, 1H), 3.52(d, *J* = 4.9 Hz, 3H), 3.29(s, 2H), 3.07–2.93(m, 9H), 2.92–2.82(m, 4H), 2.28–2.14(m, 1H),2.02(dd, *J* = 15.3, 9.7 Hz, 1H), 1.55–1.40(m, 2H), 1.35(s, 18H), 1.30–1.23(m, 2H), 0.97(m, *J* = 16.8, 10.8 Hz, 2H); ^13^C-NMR (125 MHz, DMSO-d_6_) δ/ppm = 172.25, 171.84, 171.77, 171.37, 170.22, 157.60, 156.14, 154.48, 144.47, 141.36, 138.60, 137.58, 130.90, 129.70, 129.00, 128.90, 128.39, 128.27, 128.09, 127.77, 127.32, 127.21, 125.70, 124.82, 122.92, 120.65, 120.12, 116.62, 114.99, 77.93, 73.23, 67.42, 62.14, 58.56, 58.06, 54.54, 54.25, 53.15, 52.42, 47.13, 37.79, 37.35, 36.43, 32.33, 31.43, 29.82, 28.88, 28.36, 23.02,

**Synthesis of Compound 10.** To a stirred solution of 9 (2.0 g, 1.3 mmol) in dry THF (20 mL) was added piperidine (6.5 mL, 65 mmol), dropwise, at 0 °C and the mixture was stirred at room temperature for 30 min. Solvents were evaporated off and residue was re-dissolved in dry THF (20 mL). Next, LiBr (0.5 g, 6.5 mmol) in H_2_O (1 mL) was added, dropwise, to the above solution at 0 °C followed by addition of 1M NaOH solution (6.5 mL, 6.5 mmol) over a period of 3 h. The mixture was stirred at room temperature for 12 h and concentrated in vacuo to yield 1.2 g of 10, as a white solid, in 72% yield. HRMS: *m*/*z* Calcd. 1265.6247 For C_68_H_85_N_10_O_14_, Found 1265.6262; ^1^H-NMR(600 MHz, DMSO-d_6_) δ/ppm = 10.72(s, 1H), 8.95(s, 1H), 8.74–8.63(m, 1H), 8.16(t, *J* = 9.4 Hz, 1H), 8.06(d, *J* = 7.3 Hz, 1H), 7.87(dd, *J* = 11.8, 8.0 Hz, 1H), 7.54(d, *J* = 7.9 Hz, 1H), 7.39(d, *J* = 7.2 Hz, 2H), 7.37–7.32(m, 2H), 7.32–7.28(m, 1H), 7.29–7.14(m, 10H), 7.14–6.98(m, 4H), 6.92–6.88(m, 1H), 6.85(d, *J* = 8.3 Hz, 2H), 6.79(dd, *J* = 18.0, 7.3 Hz, 1H), 6.68(t, *J* = 5.7 Hz, 1H), 5.56(d, *J* = 40.3, 8.0 Hz, 1H), 5.00(s, 2H), 4.54(dq, *J* = 35.6, 7.4 Hz, 1H), 4.46–4.34(m, 2H), 4.07(s, 2H), 3.09–2.88(m, 9H), 2.74(dt, *J* = 31.7, 10.3 Hz, 3H), 2.64(d, *J* = 15.3 Hz, 1H), 2.55(d, *J* = 15.3 Hz, 1H), 2.26(d, *J* = 44.3 Hz, 1H), 1.94–1.83(m, 1H), 1.46–1.37(m, 1H), 1.36–1.31(m, 18H), 1.27(t, *J* = 7.6 Hz, 1H), 1.19(dt, *J* = 17.5, 8.5 Hz, 2H), 0.98–0.79(m, 2H); ^13^C-NMR(150 MHz, DMSO-d_6_) δ/ppm = 173.02, 171.60, 171.16, 169.77, 157.28, 156.09, 139.56, 139.27, 138.16, 137.58, 136.65, 136.48, 130.59, 130.15, 130.05, 128.81, 128.72, 128.12, 128.03, 127.81, 127.06, 125.98, 124.16, 121.24, 118.99, 118.65, 114.75, 111.70, 110.77, 110.38, 77.77, 76.08, 69.51, 59.74, 55.92, 55.35, 55.10, 53.90, 53.07, 38.35, 37.75, 37.13, 32.22, 31.65, 29.59, 28.68, 27.97, 23.02.

**Synthesis of intermediate A.** To a stirred solution of 10 (1.0 g, 0.8 mmol) in dry DMF (250 mL) was added HATU (0.8 g, 2.0 mmol) and anhydrous HOBt (0.3 g, 2.0 mmol), slowly, and the mixture was stirred at −5 °C for 2 h. After the reaction was completed, H_2_O (20 mL) was added to the above solution and white solid precipitated out. The mixture was filtered through celite and the solid residue was obtained to give 0.86 g of intermediate A in 87% yield. HRMS: *m*/*z* Calcd. 1247.6141 For C_68_H_83_N_10_O_13_, Found 1247.6098; ^1^H-NMR(600 MHz, DMSO-d_6_) δ/ppm = 10.85(s, 1H), 9.34(d, *J* = 7.4 Hz, 1H), 8.33(d, *J* = 6.8 Hz, 1H), 7.90(d, *J* = 12.6 Hz, 1H), 7.71(d, *J* = 9.8 Hz 1H), 7.55(d, *J* = 16.2 Hz, 1H), 7.43(d, *J* = 18.4 Hz, 2H), 7.33(ddt, *J* = 36.4, 21.3, 7.4 Hz, 10H), 7.22(dd, *J* = 15.1, 7.6 Hz, 3H), 7.19–7.14(m,1H), 7.06–6.91(m, 3H), 6.88(d, *J* = 6.5 Hz, 1H), 6.83–6.74(m, 3H), 6.72–6.61(m, 1H), 5.39(d, *J* = 8.3 Hz, 1H), 5.18(s, 1H), 5.05–4.92(m, 3H), 4.60–4.46(m, 1H), 4.49–4.32(m, 2H), 3.69(dd, *J* = 34.6, 7.6 Hz, 1H), 3.62–3.51(m, 1H), 3.04–2.85(m, 7H), 2.83–2.55(m, 5H), 2.15(d, *J* = 7.0 Hz, 1H), 2.08–1.89(m, 1H), 1.44(s, 1H), 1.33(s, 18H), 1.26–1.08(m, 3H), 1.03–0.86(m, 2H); ^13^C-NMR(150 MHz, DMSO-d_6_) δ/ppm = 172.60, 171.70, 171.17, 169.87, 157.41, 156.06, 139.95, 137.64, 137.56, 136.76, 136.45, 136.41, 131.19, 130.67, 130.28, 129.73, 129.31, 129.16, 128.85, 128.50, 128.24, 128.12, 127.43, 126.28, 124.21, 121.27, 118.62, 114.50, 111.65, 109.67, 78.22, 77.89, 69.58, 59.91, 57.41, 54.89, 53.76, 51.74, 51.11, 36.62, 36.29, 31.70, 30.95, 29.54, 28.69, 27.95, 22.98.

**Synthesis of pasireotide.** TFA (6.0 mL) was added to intermediate A (0.5 g, 0.4 mmol) and the mixture was stirred at 0 °C for 2 h. After the reaction was completed, excess TFA was evaporated off. Diethyl ether was added to the residue and a white solid was precipitated off to give 0.45 g of pasireotide after filtration (89% yield). ESI-MS(*m*/*z*): 524.04[M/2 + H−CF_3_COOH]^+^; 1047.86[M + H−2CF_3_COOH]^+^; 1069.81[M + Na−2CF_3_COOH]^+^; HRMS: *m*/*z* Calcd. 1047.5092 For C_58_H_67_N_10_O_9_, Found 1047.5107; ^1^H-NMR(600 MHz, DMSO-d_6_) δ/ppm = 10.87(s, 1H), 7.82(d, *J* = 42 Hz, 10H), 7.42–7.38(m, 5H), 7.36–7.25(m, 10H), 7.23–7.21(t, *J* = 6.0 Hz, 3H), 7.07–7.04(t, *J* = 9.0 Hz, 1H), 7.0(s, 1H), 6.96–6.93(t, *J* = 9.0 Hz, 3H), 6.82(d, *J* = 12 Hz, 2H), 5.48(d, *J* = 6.0 Hz, 1H), 5.22(s, 1H), 5.01(s, 2H), 4.59(d, *J* = 6.0 Hz, 1H), 4.46–4.43(dd, *J* = 10.2, 7.0 Hz, 1H), 4.41–4.39(m, 1H), 3.98(s, 1H), 3.78(d, *J* = 12.0 Hz, 1H), 3.70(q, *J* = 6.0 Hz, 1H), 3.60(d, *J* = 6.0 Hz, 1H), 3.28–3.24(m, 2H), 3.22–3.16(m, 2H), 3.03–3.01(m, 2H), 2.90–2.84(m, 4H), 2.69(d, *J* = 12.0 Hz, 2H), 2.20(d, *J* = 6.0 Hz, 1H), 2.05–2.01(t, *J* = 12.0 Hz, 1H), 1.43–1.27(m, 2H), 1.22–1.17(m, 2H), 0.92(s, 1H), 0.72(s, 1H); ^13^C-NMR (150 MHz, DMSO-d_6_) δ/ppm = 172.57, 170.58, 170.30, 170.10, 168.90, 157.33, 156.03, 138.32, 137.61, 137.01, 136.43, 130.50, 130.32, 130.27, 128.92, 128.88, 128.50, 128.26, 128.20, 128.15, 128.08, 127.04, 124.29, 121.30, 120.68, 118.88, 118.70, 116.71, 114.80, 111.70, 109.77, 74.13, 69.60, 59.97, 57.72, 57.55, 56.71, 56.14, 54.79, 53.11, 40.52, 39.12, 38.96, 38.45, 37.54, 36.25, 34.49, 31.09, 26.93, 22.22.

## Figures and Tables

**Table 1:** Synthesis of protected peptides via BSA/NHS method.

Entry	Product	Time (h)	Solvent	BSA/AA/NHS Ester ^a^	Yield (%) ^b^
1	compound **2**	72 h	CH_2_Cl_2_	-/1.2/1.0	Trace
2	compound **2**	12 h	CH_2_Cl_2_	2.1/1.2/1.0	82% ^c^
3	compound **2**	12 h	CH_2_Cl_2_	1.1/1.2/1.0	62%
4	compound **2**	6 h	CH_2_Cl_2_	4.4/1.2/1.0	53%
5	fragment B	12 h	THF	2.1/1.2/1.0	84% ^c^
6	fragment B	12 h	THF	1.1/1.2/1.0	65%
7	fragment B	12 h	THF	4.4/1.2/1.0	51%
8	fragment B	12 h	CH_2_Cl_2_	2.2/1.2/1.0	85%

^a^ The molar ratio of BSA to amino acid (AA) and NHS ester; AA = *Ne*-Boc-l-lysine for compound **2**; AA = the amine intermediate for fragment B. ^b^ Isolated yield. ^c^ Better yield after optimization conditions.

**Table 2:** Hydrolysis of compound **9**.

Entry	Conditions	Racemic Product
1	NaOH, THF	Yes
2	LiOH, THF/MeOH	Yes
3	LiBr/NaOH, THF	No

## Supplementary Materials

The following are available online.
