# Supplementary material for: A Practical and Total Synthesis of Pasireotide: Synthesis of Cyclic Hexapeptide via a Three-Component Condensation
Source: Molecules. 2019 Jun 11;24(11):2185. doi: 10.3390/molecules24112185 (PMC6600510; doi:10.3390/molecules24112185)
Supplement: Supplementary file 1 [file molecules-24-02185-s001.pdf]

A practical and total synthesis of pasireotide: synthesis of cyclic hexapeptide via a three-component condensation

Chunying Ma, Miao Chen, Weiming Chu, Jiayi Tao, Delong Kong, Mengmeng Zhang, Wenhua Feng

Department of New Drug Research and Development, Institute of Materia Medical, Chinese Academy of Medical Sciences & Peking Union Medical College, Beijing 100050, China

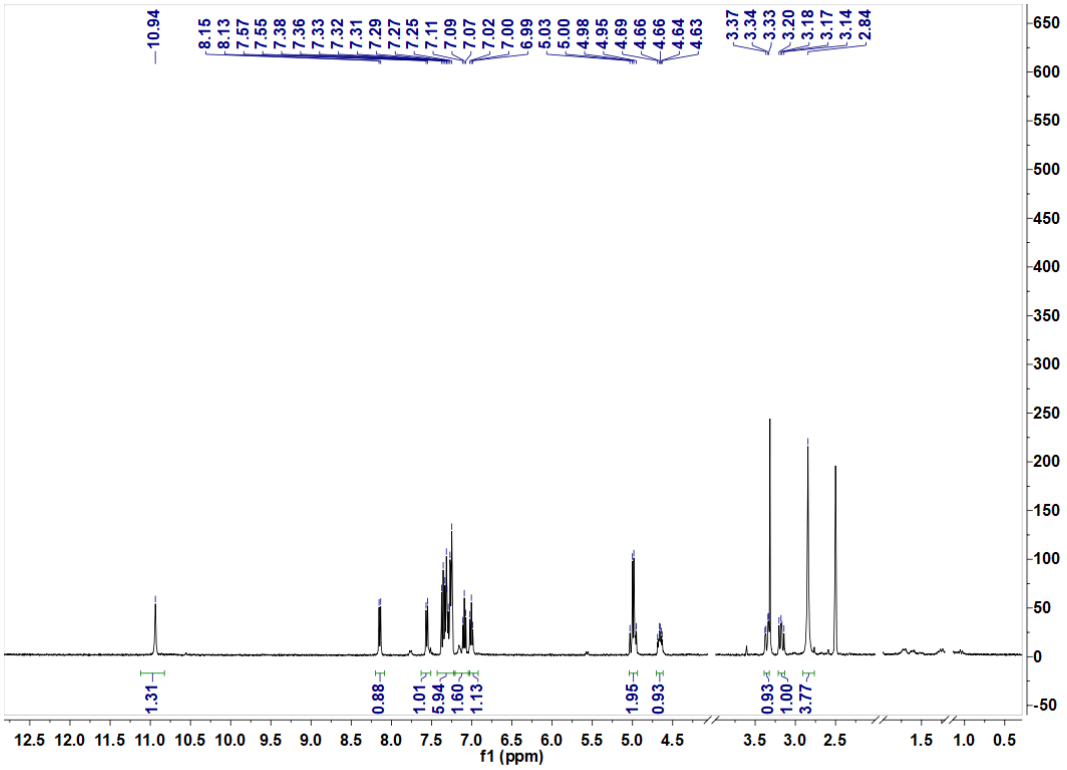

Figure S1. ^{1}H-NMR spectra of compound 1.

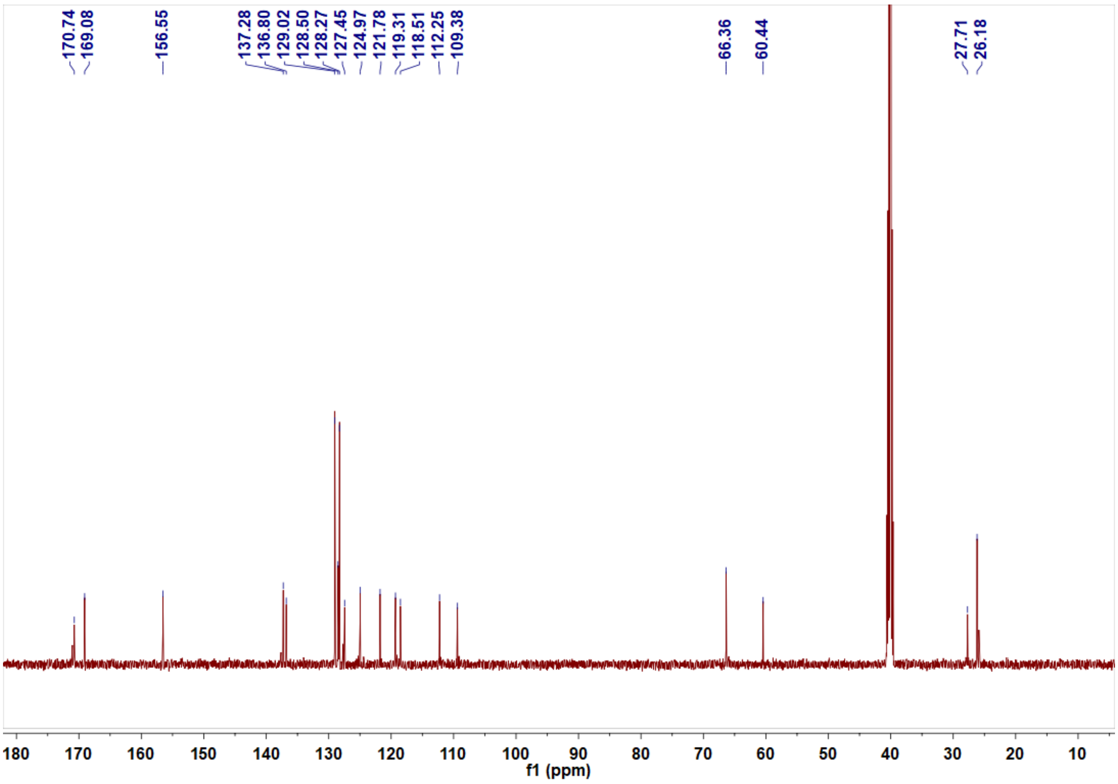

Figure S2. ^{13}C-NMR spectra of compound 1.

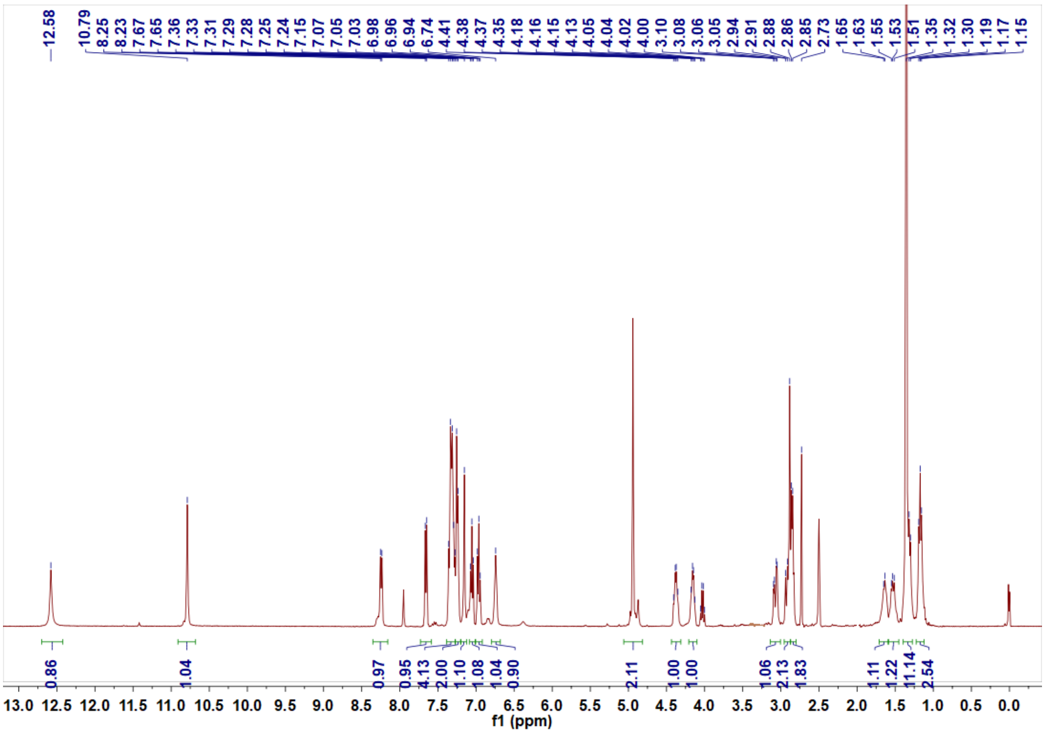

Figure S3. ^{1}H-NMR spectra of compound 2.

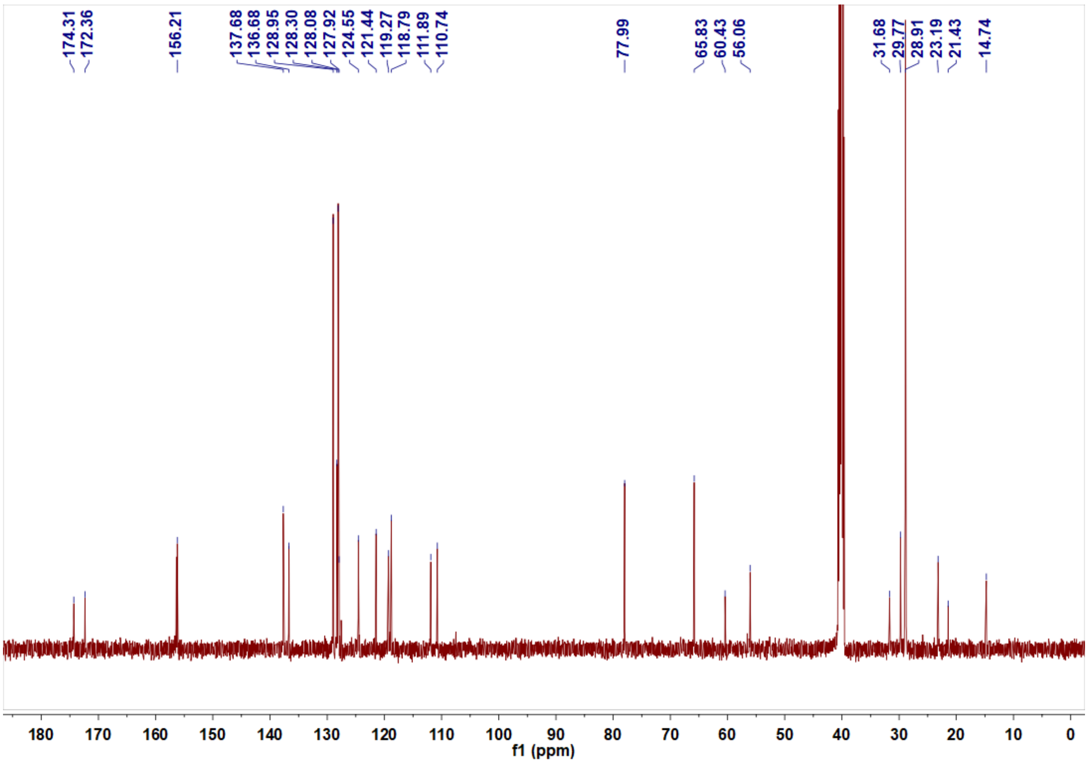

Figure S4. ^{13}C-NMR spectra of compound 2.

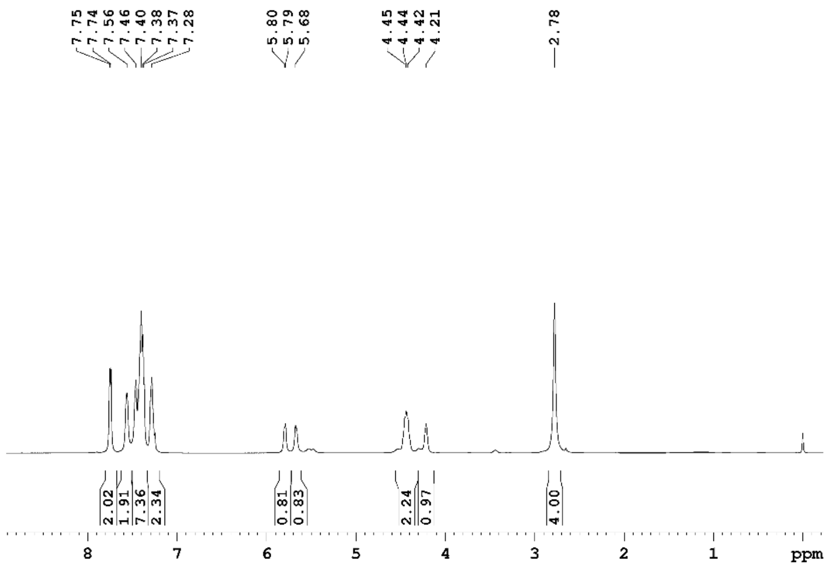

Figure S5. ^{1}H-NMR spectra of compound 3.

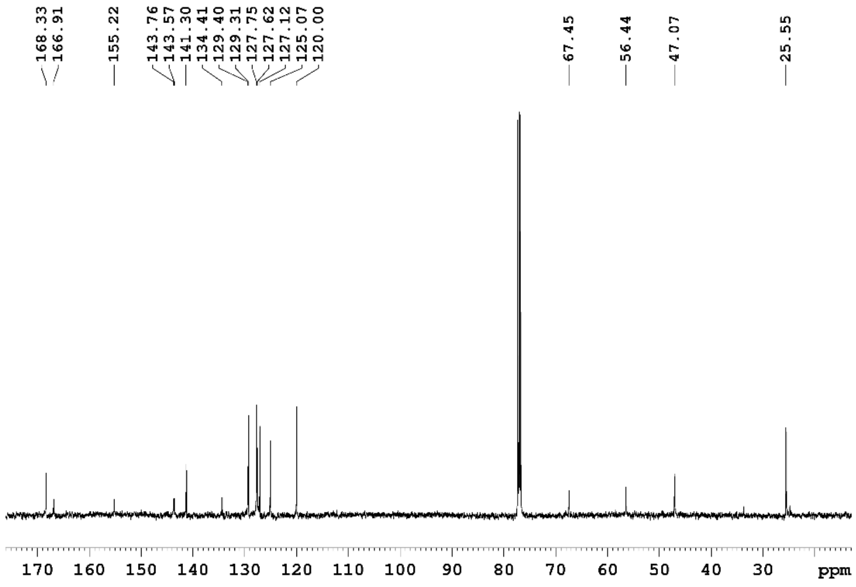

Figure S6. ^{13}C-NMR spectra of compound 3.

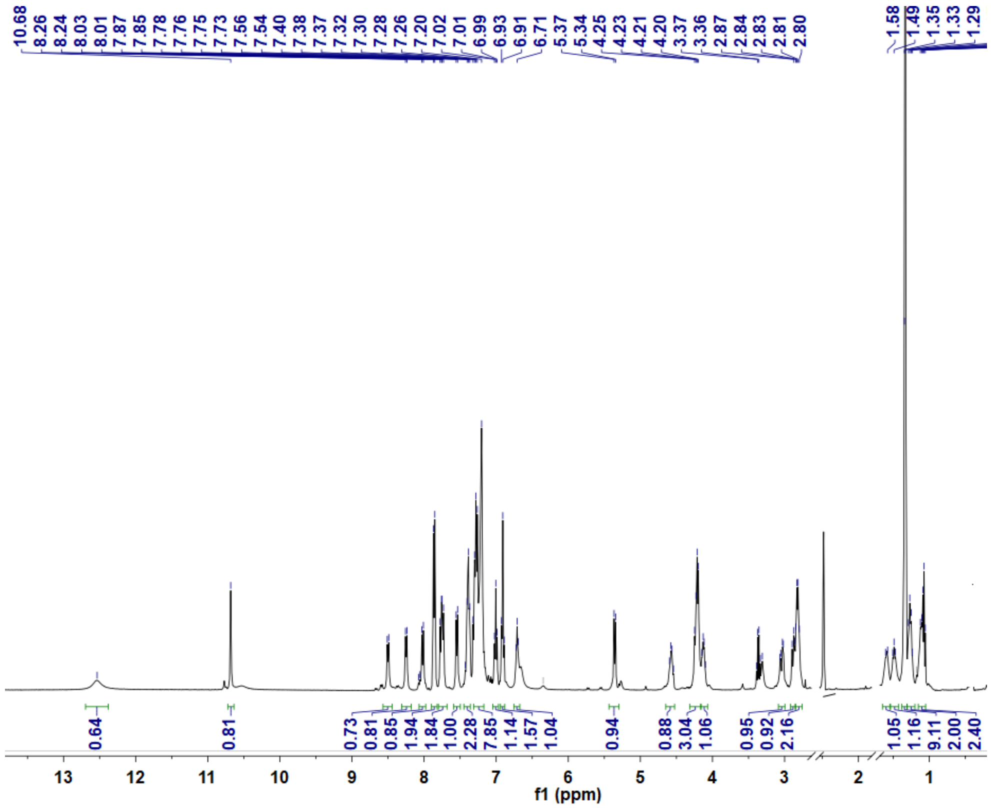

Figure S7. ^{1}H-NMR spectra of fragment B.

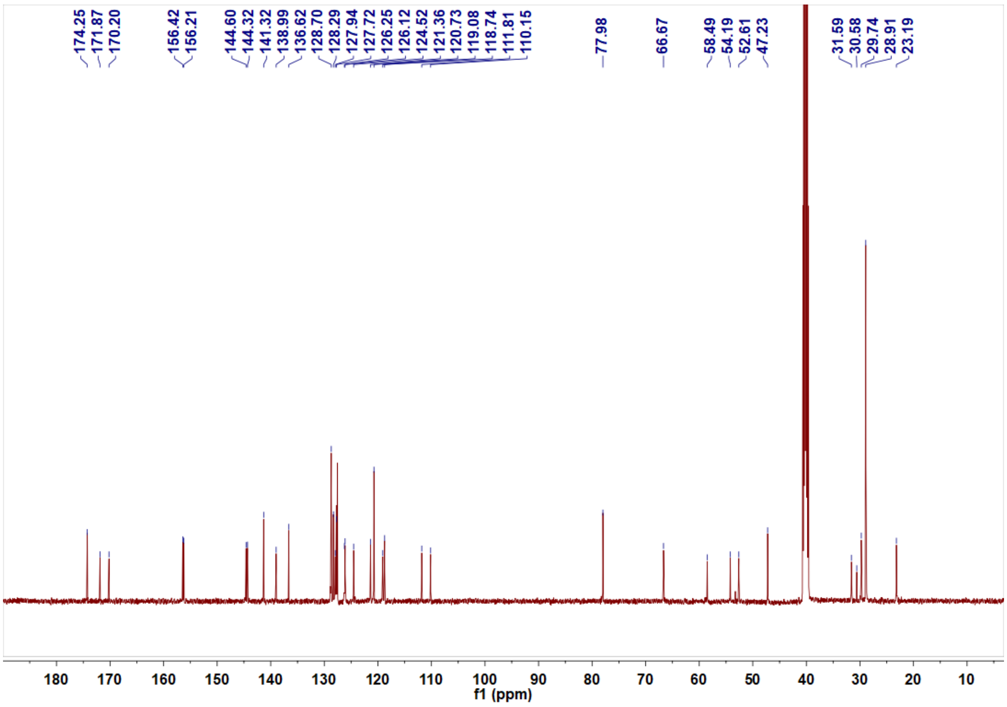

Figure S8. ^{13}C-NMR spectra of fragment B.

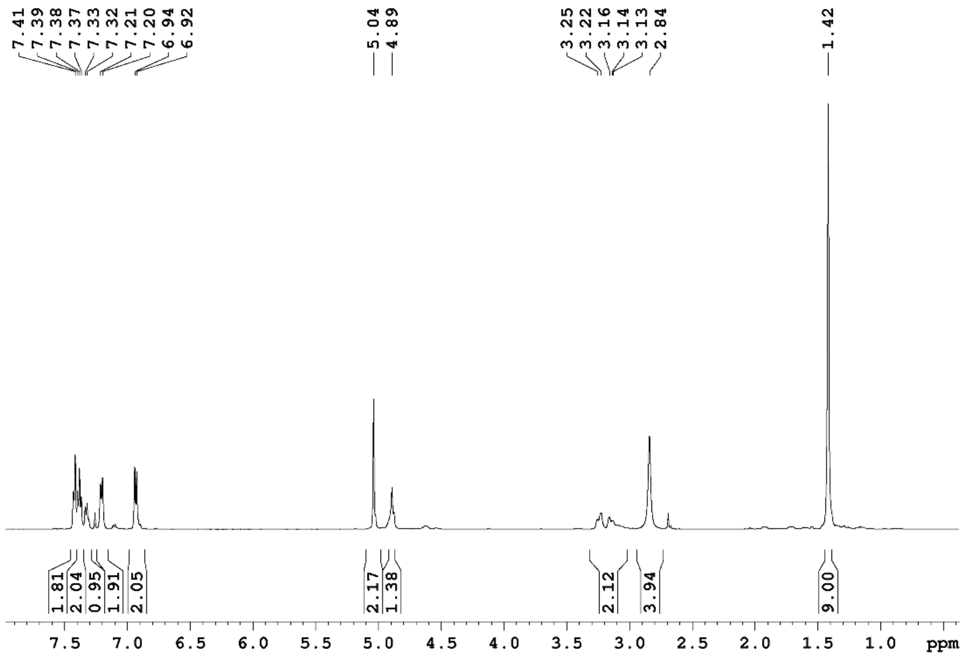

Figure S9. ^{1}H-NMR spectra of compound 8.

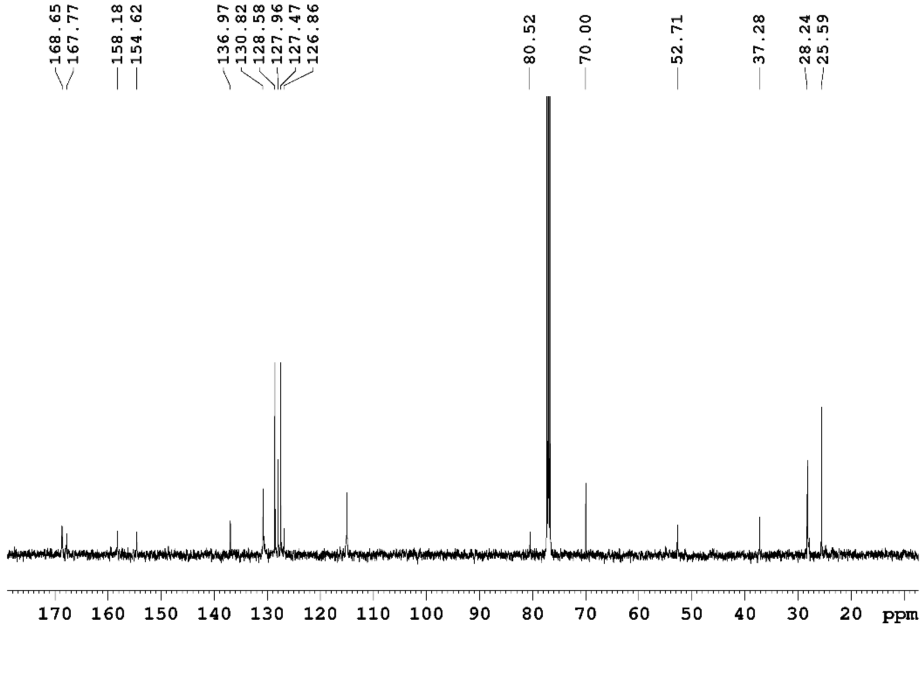

Figure S10. ^{13}C-NMR spectra of compound 8.

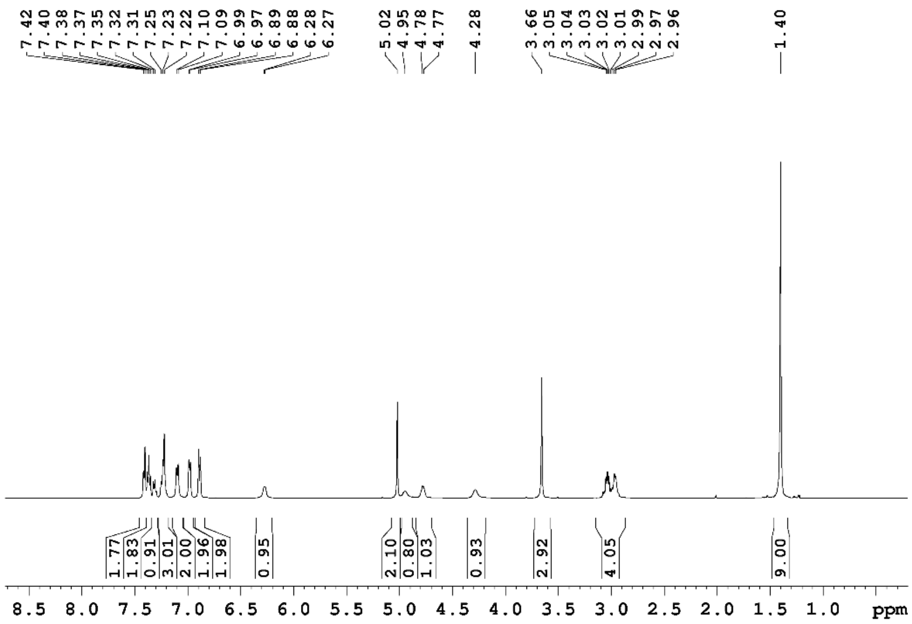

Figure S11. ^{1}H-NMR spectra of compound 5.

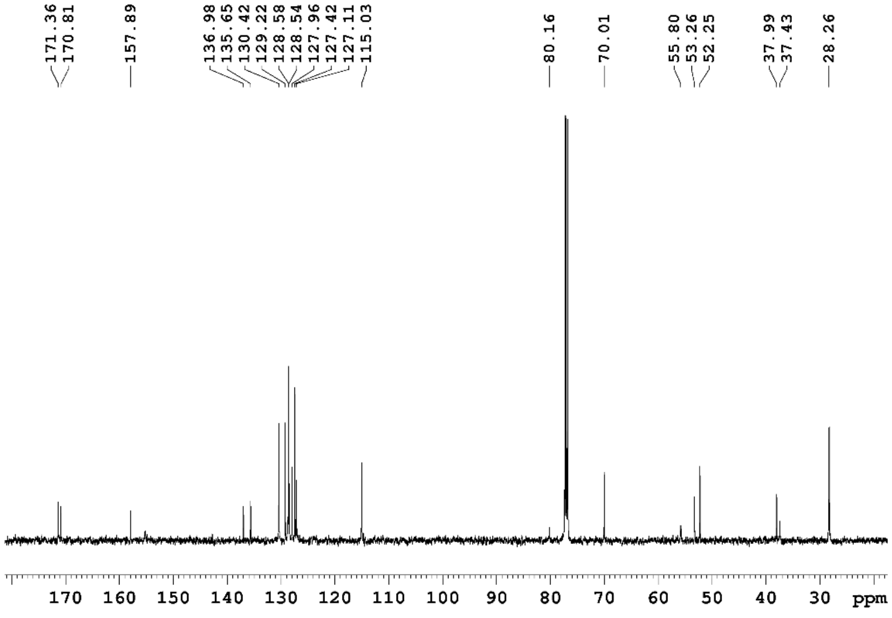

Figure S12. ^{13}C-NMR spectra of compound 5.

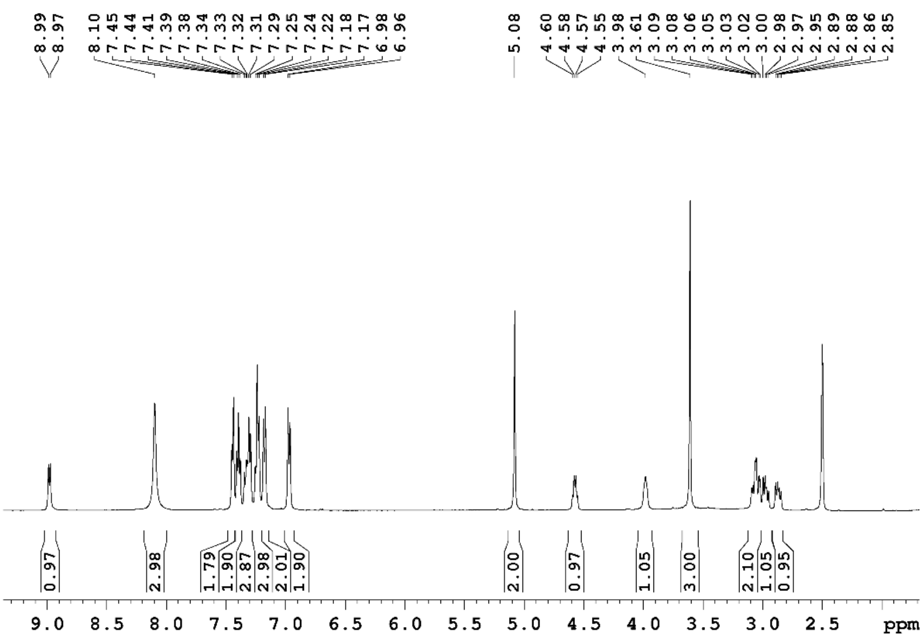

Figure S13. ^{1}H-NMR spectra of fragment C.

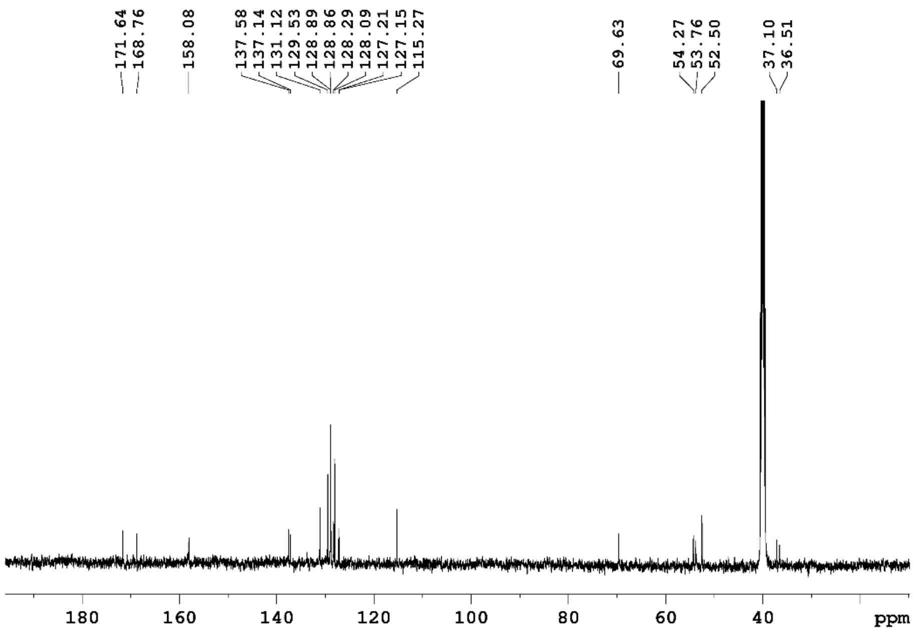

Figure S14. ^{13}C-NMR spectra of fragment C.

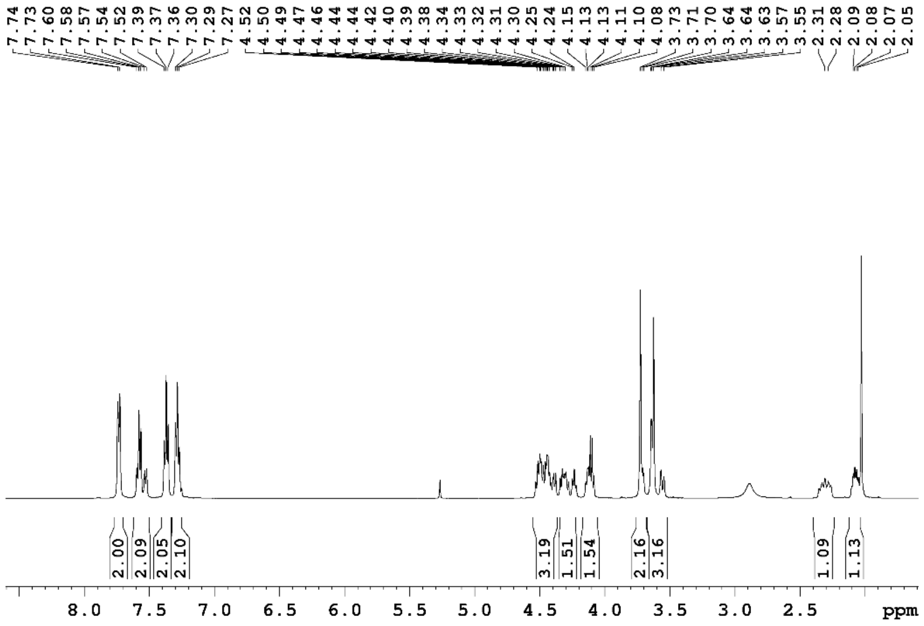

Figure S15. ^{1}H-NMR spectra of compound 6.

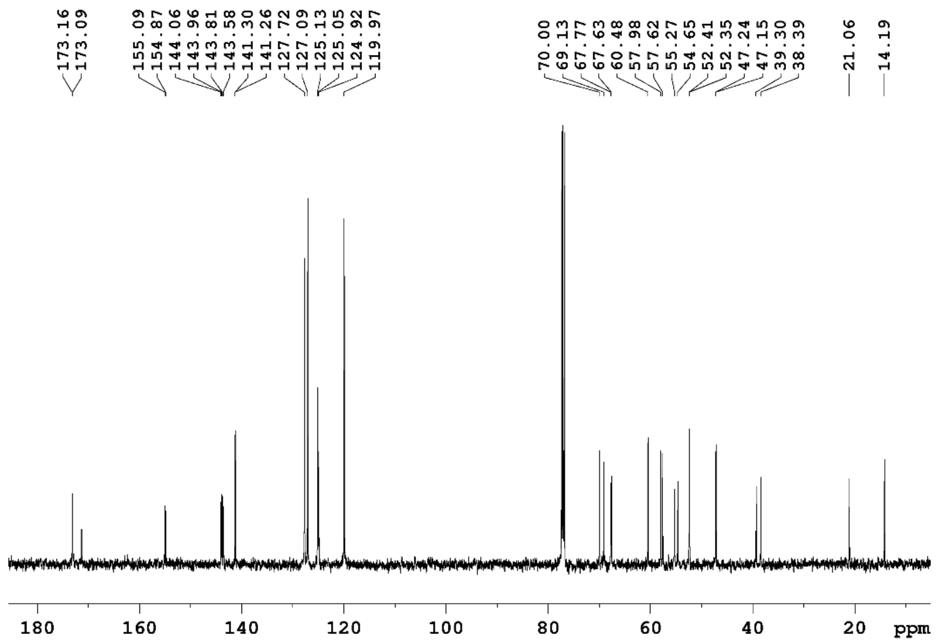

Figure S16. ^{13}C-NMR spectra of compound 7.

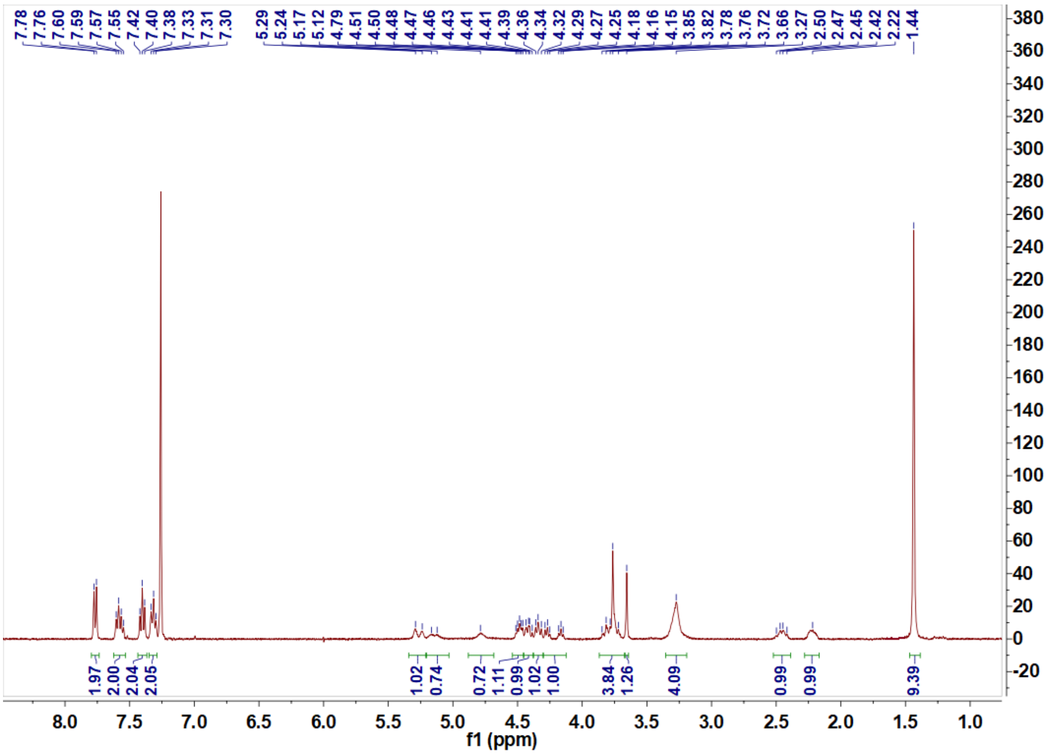

Figure S17. ^{1}H-NMR spectra of compound 7.

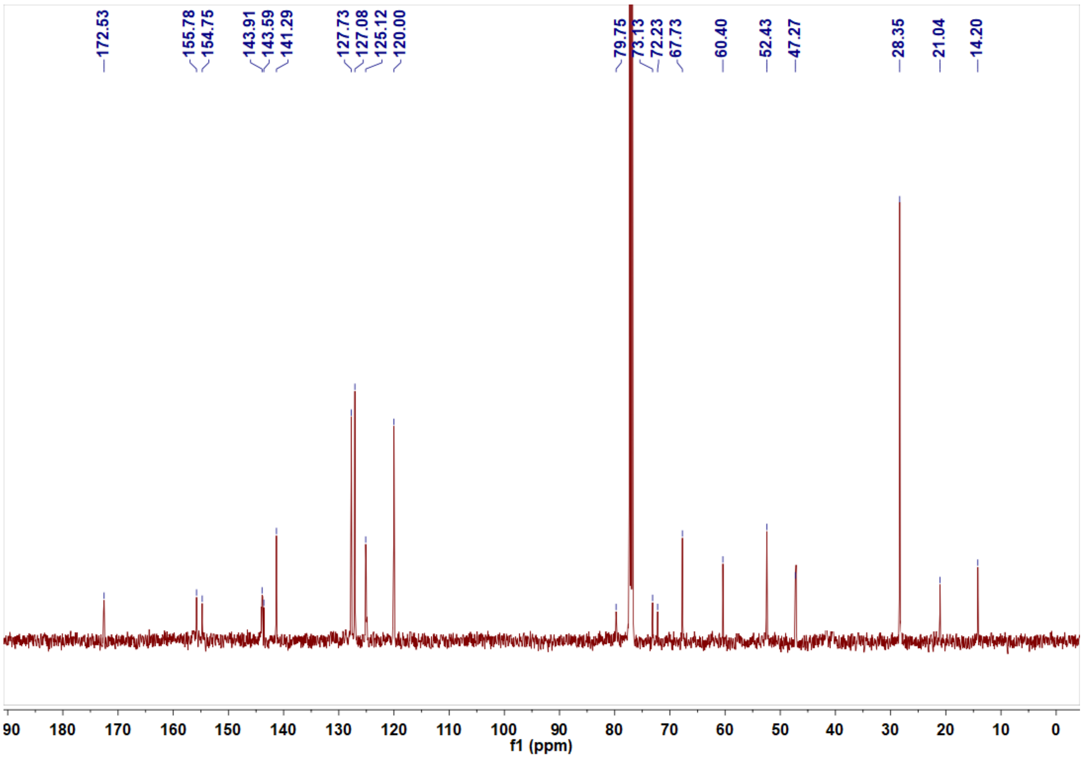

Figure S18. ^{13}C-NMR spectra of compound 7.

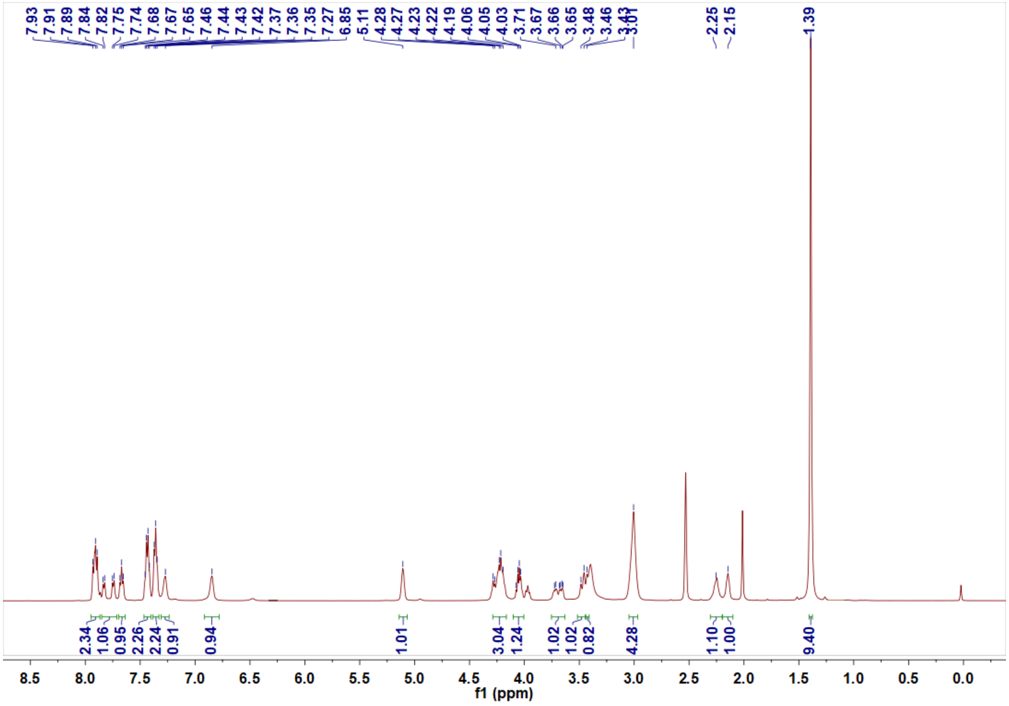

Figure S19. ^{1}H-NMR spectra of fragment D.

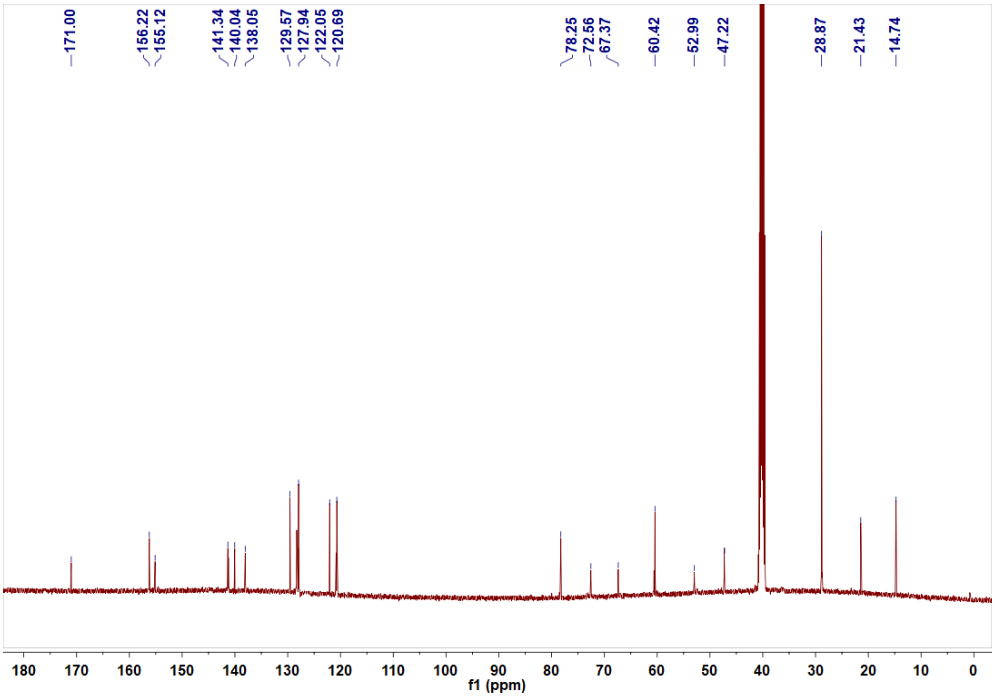

Figure S20. ^{13}C-NMR spectra of fragment D.

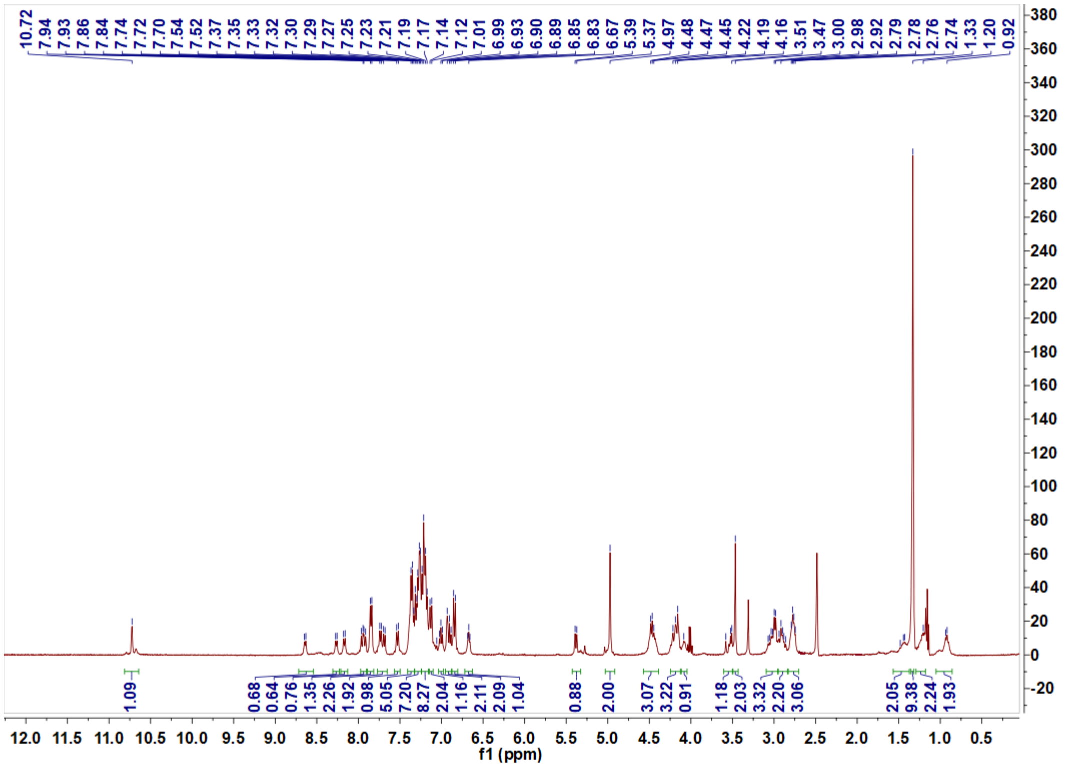

Figure S21. ^{1}H-NMR spectra of compound 8.

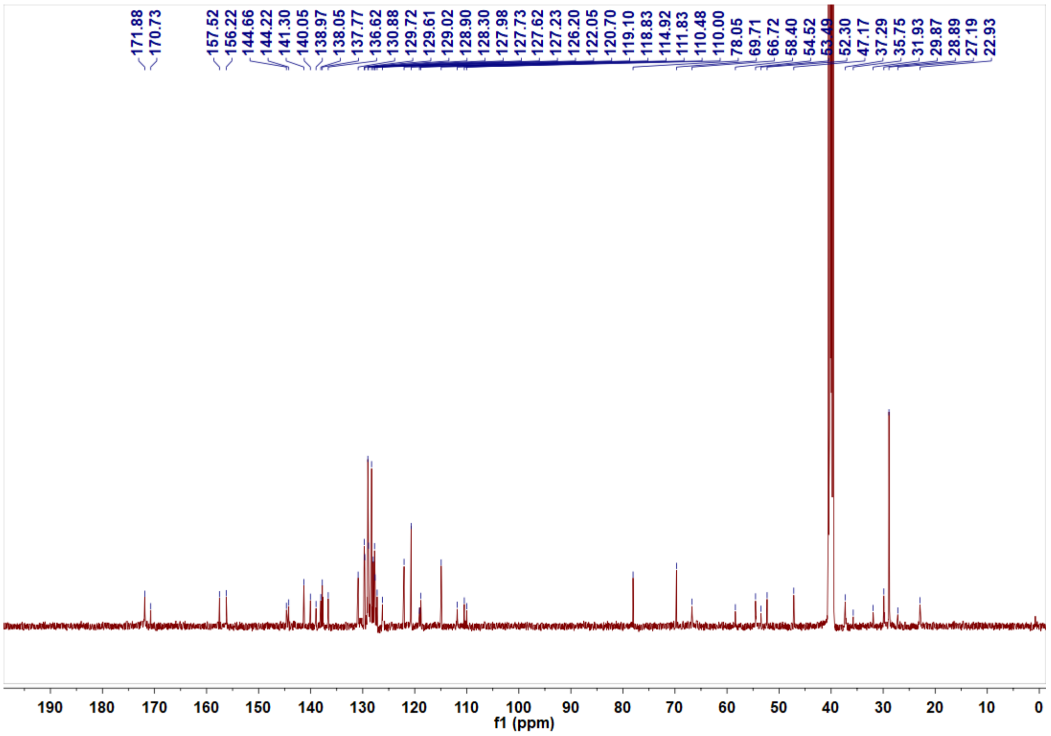

Figure S22. ^{13}C-NMR spectra of compound 8.

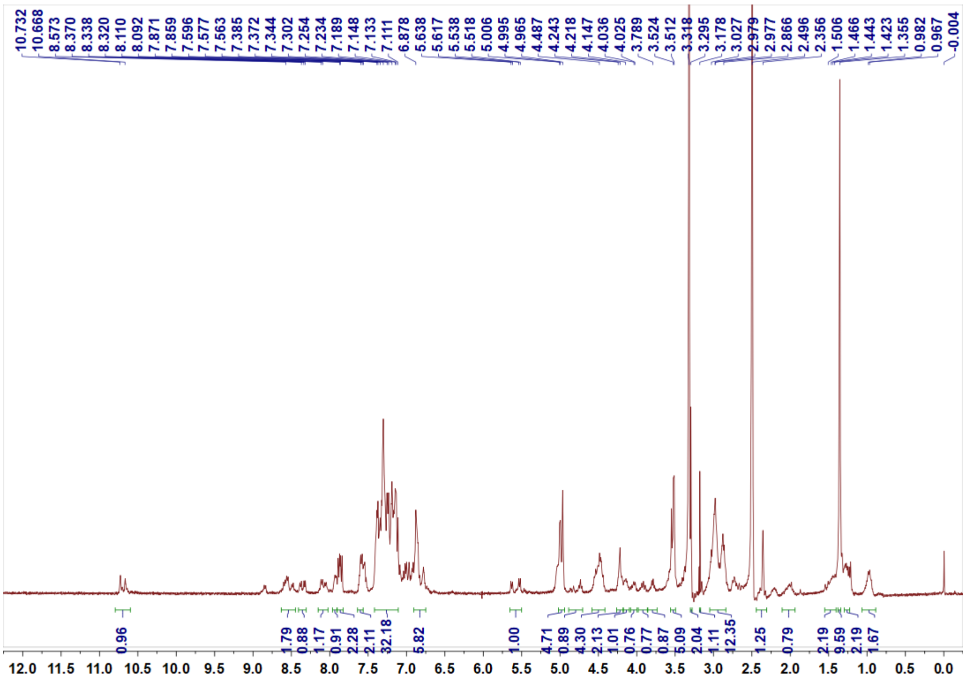

Figure S23. ^{1}H-NMR spectra of compound 9.

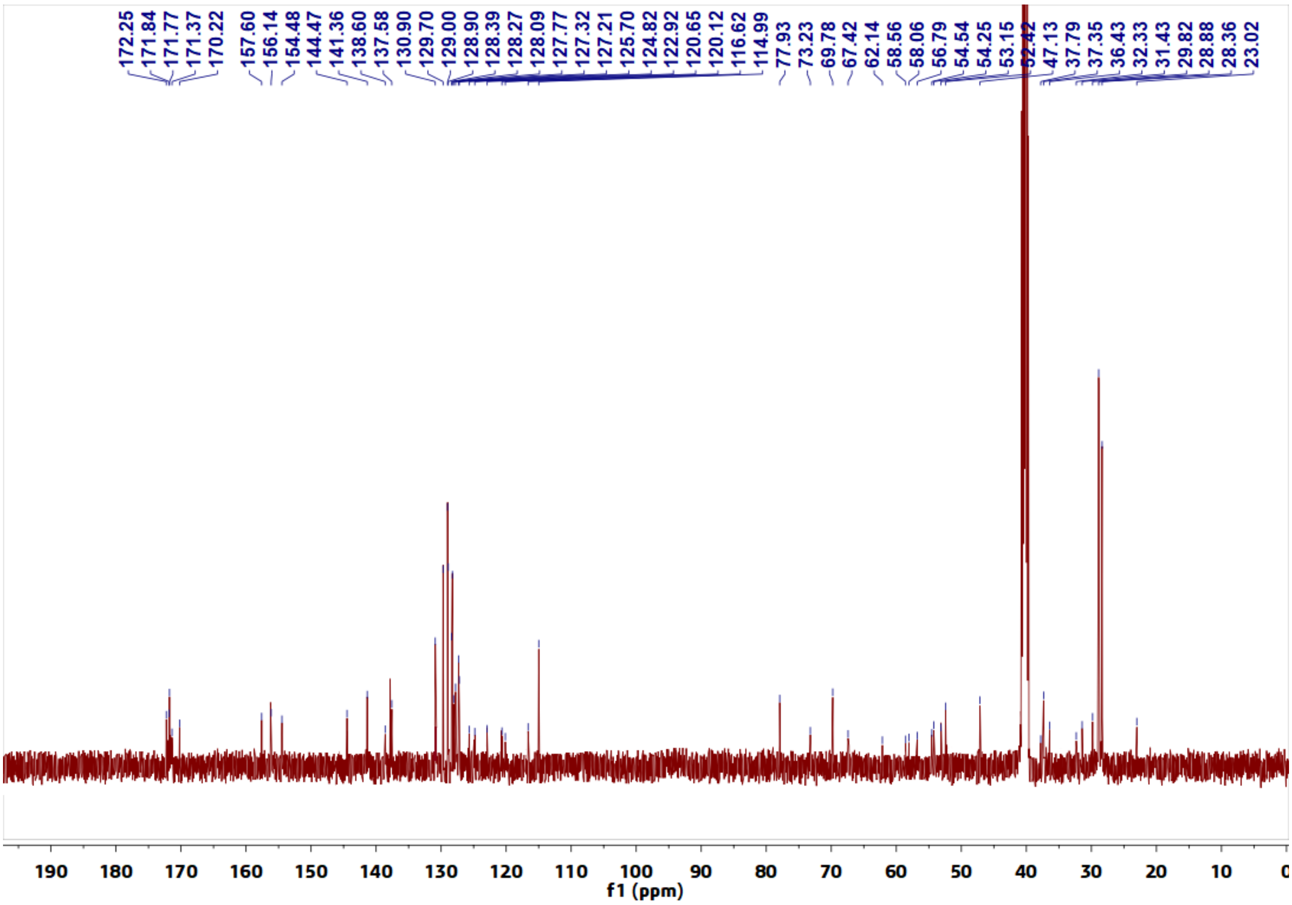

Figure S24. ^{13}C-NMR spectra of compound 9.

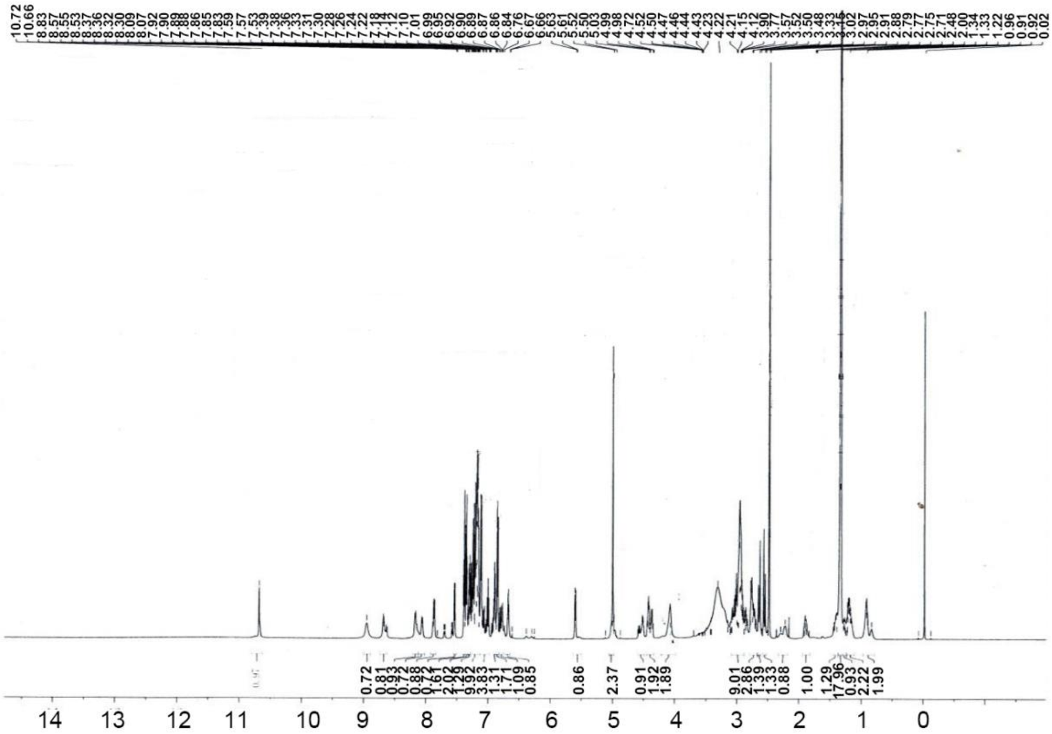

Figure S25. ^{1}H-NMR spectra of compound 10.

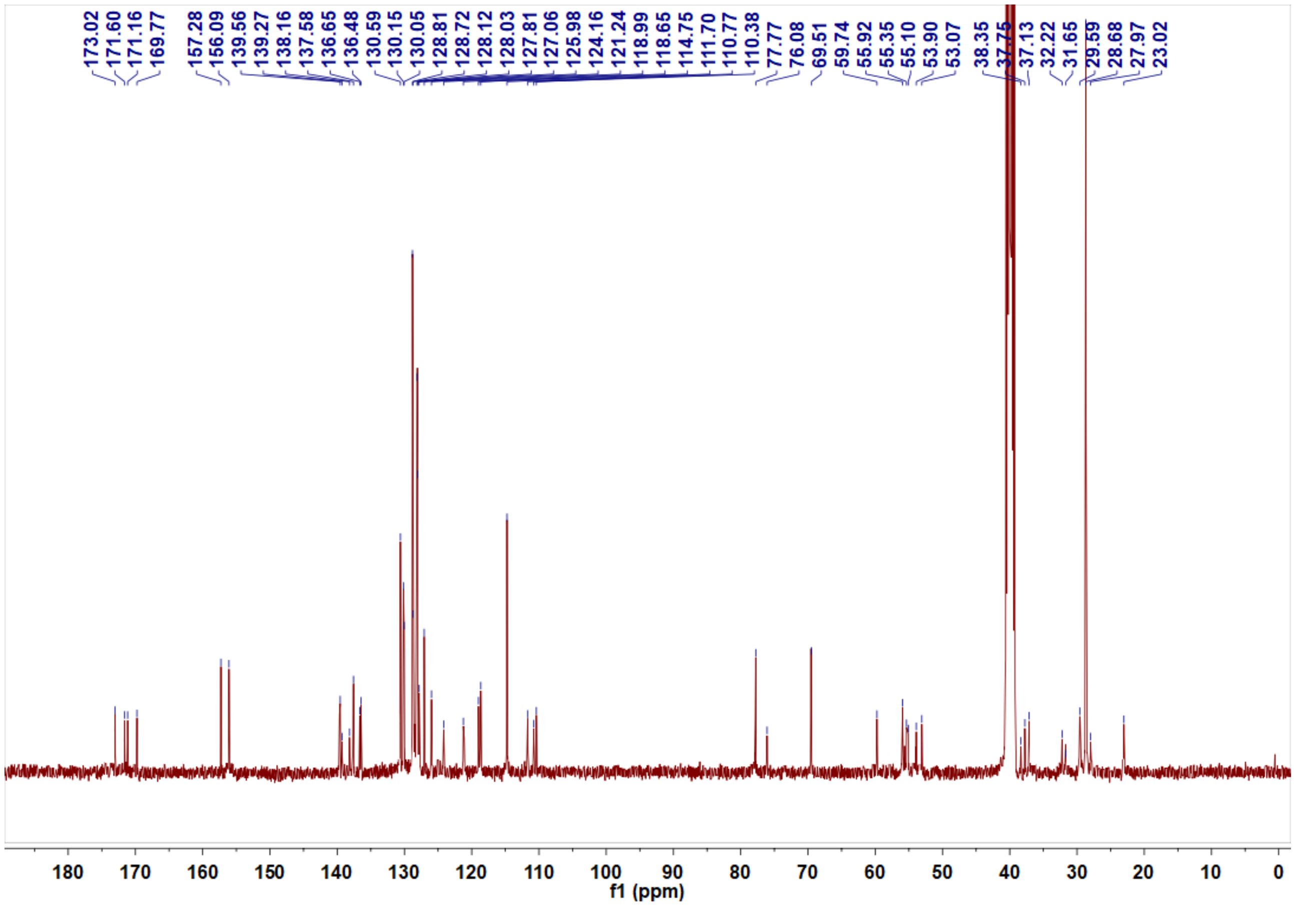

Figure S26. ^{13}C-NMR spectra of compound 10.

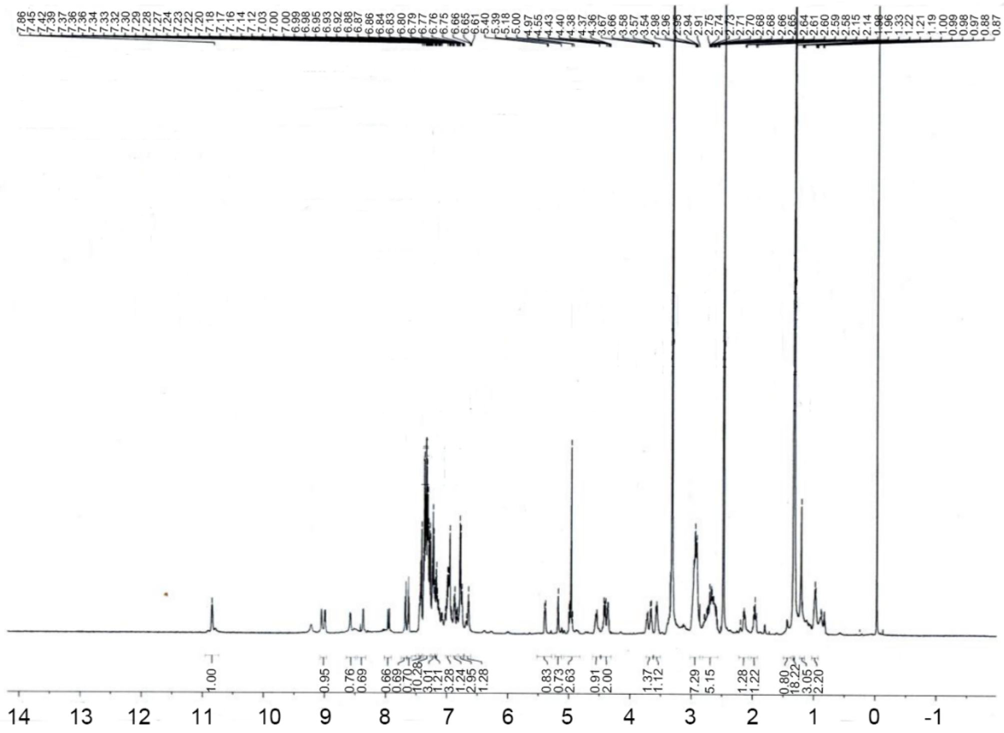

Figure S27. ^{1}H-NMR spectra of intermediate A.

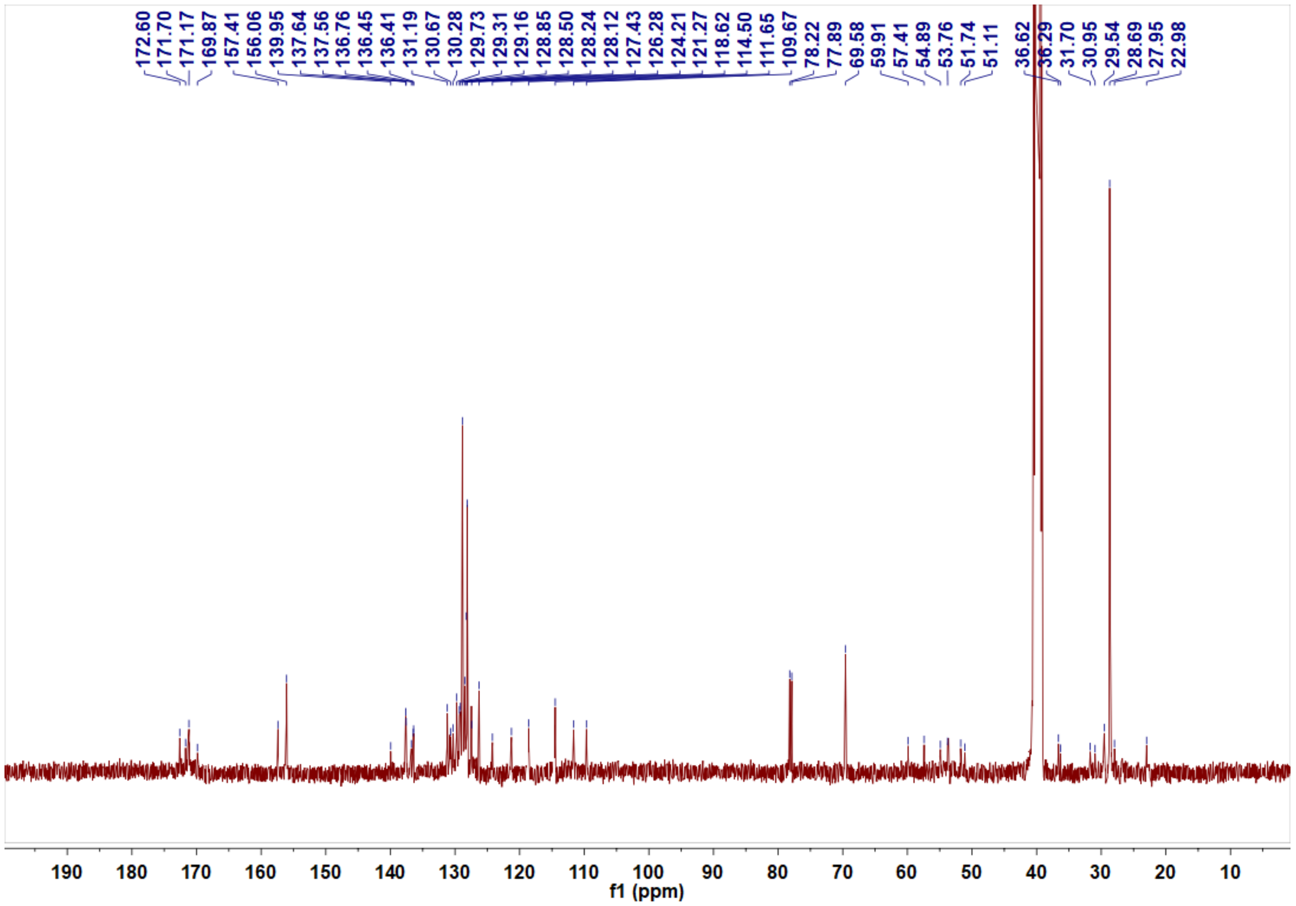

Figure S28. ^{13}C-NMR spectra of intermediate A.

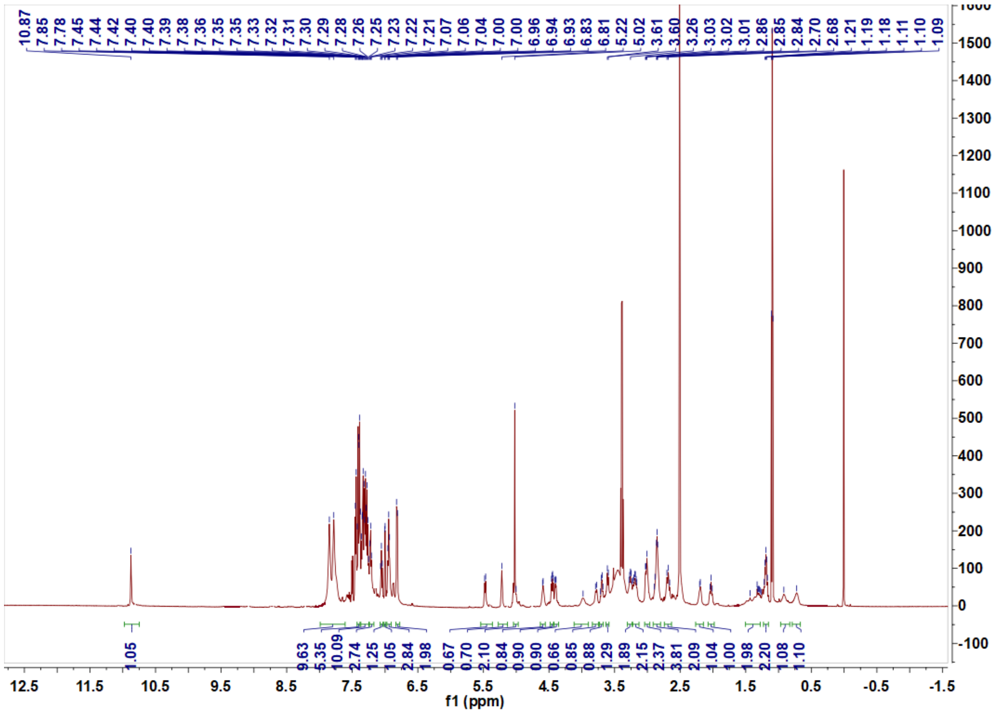

Figure S29. ^{1}H-NMR spectra of pasireotide.

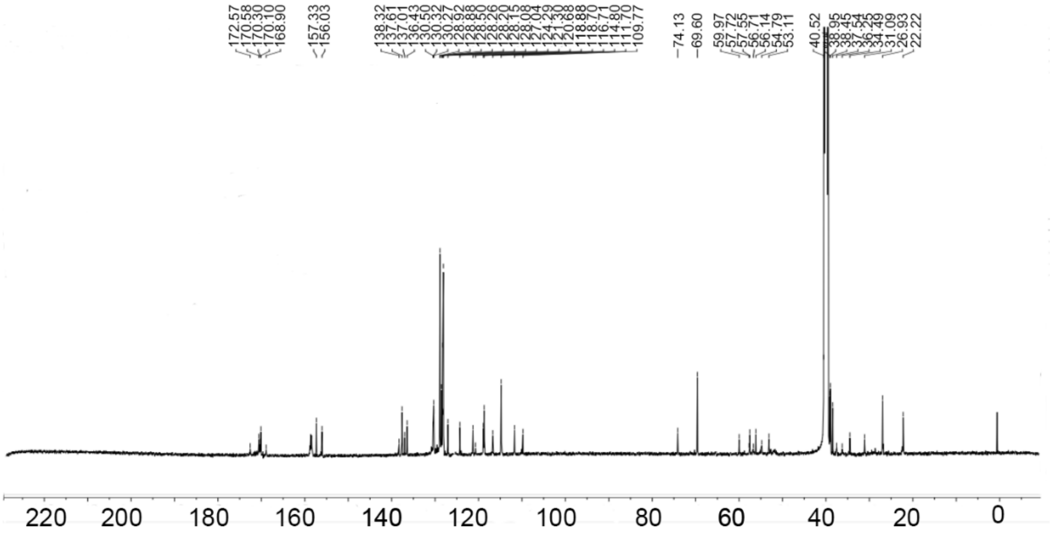

Figure S30. ^{13}C-NMR spectra of pasireotide.

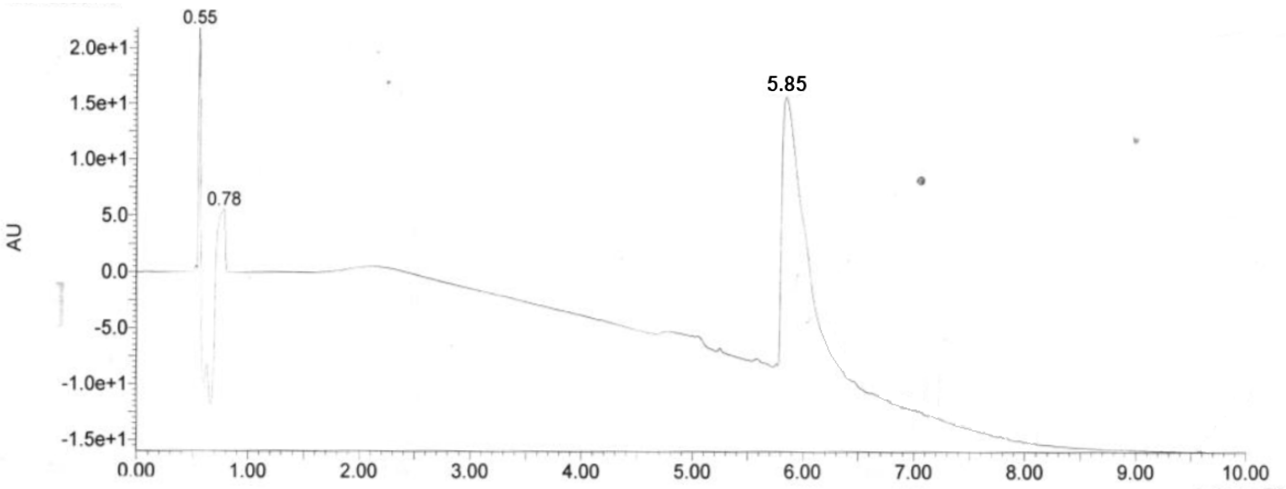

Figure S31. HPLC spectra of compound 18 after LiBr/NaOH hydrolysis conditions.

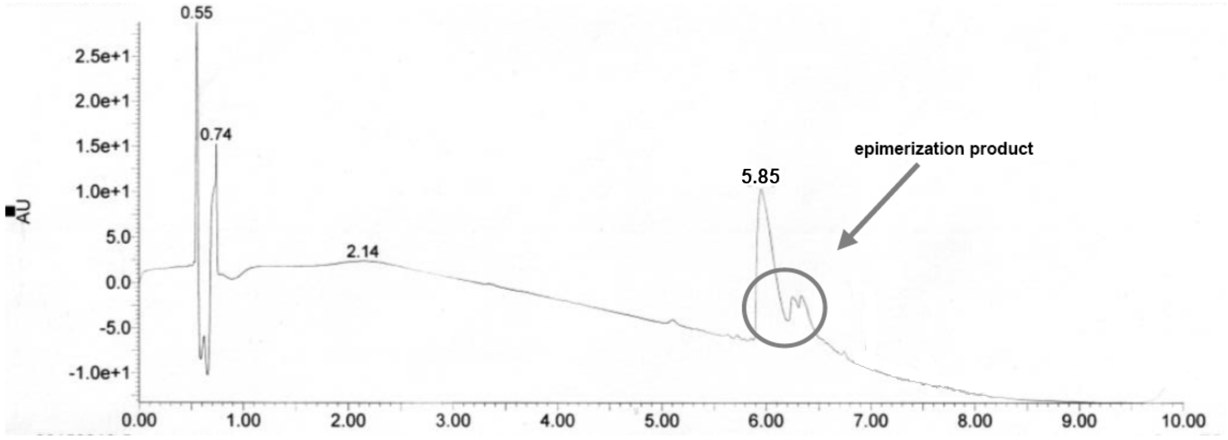

Figure S32. HPLC spectra of compound 18 after NaOH hydrolysis conditions.

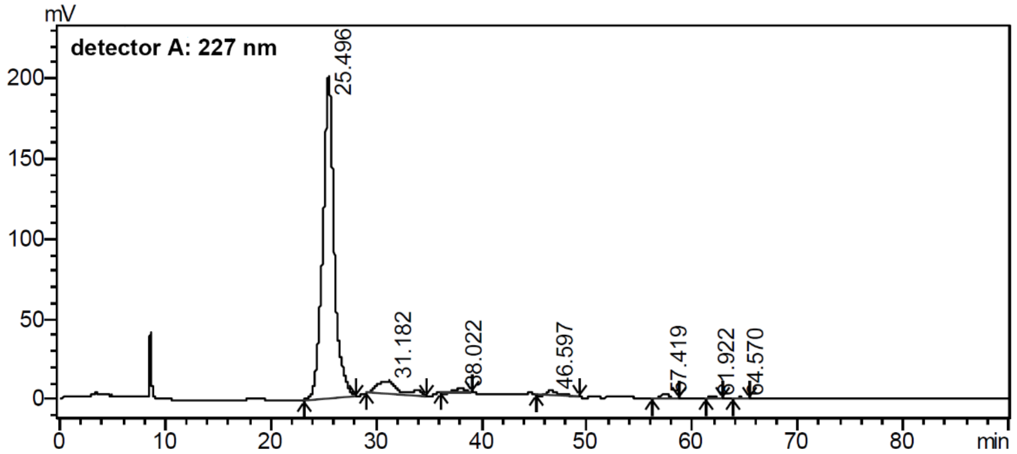

Figure S33. preparative reversed-phase HPLC spectra of pasireotide.

Table S1. Cyclization conditions：coupling reagents, reaction temperature, time, and reagent concentration.

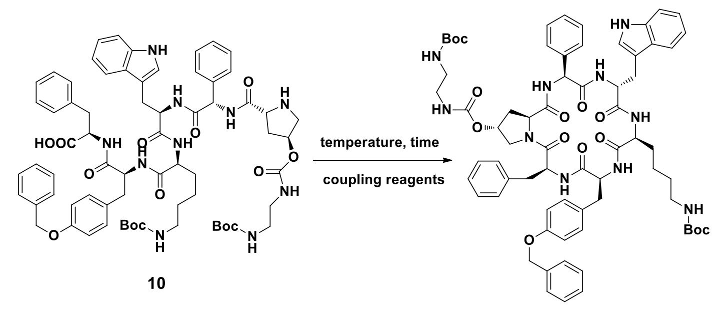

Entry	Coupling reagent	Temperature(°C)	Time(h)	Reagent concentration (mM)	Yield (%)
1	DCC/HOBT	0	2	64	30
2	BOP/DMAP	0	2	64	47
3	TBTU/4-methylmorpholine	0	2	64	69
4	TBTU/4-methylmorpholine	-5	6	64	71
4	HATU/HOBt	0	2	64	80
5	HATU/HOBt	-5	2	32	87
6	HATU/HOBt	25	2	1	69

Table S2. Gradient elution conditions.

Compound	Chromatographic column	Time (min)	A (%)	B (%)
fragment B	Agilent Eclipse XDB-C18, 4.6 × 250 nm, 5*u*M	0	20	80
		30	80	20
		31	20	80
		40	20	80
fragment C	Agilent Extend-C18, 4.6 × 250 mm, 5*u*M	0	45	55
		15	70	30
		30	75	25
		35	75	25
		40	45	55
		45	45	55
fragment D	Agilent Extend-C18, 4.6 × 250 mm, 5*u*M	0	35	65
		30	50	50
		45	80	20
		50	80	20
		55	35	65
10	Waters UPLC BEH C18, 2.1 × 50 mm, 17uM	0	5	95
		6	95	5

		8	95	5
		50	5	95
crude parireotide	SHIMADZU Shim-pack PRC-ODS (H), 20 mm × 250 mm	0	5	95
		20	95	5
		50	95	5
		50.1	5	95
		70	5	95
